# A Latent Pro-Survival Function for the Mir-290-295 Cluster in Mouse Embryonic Stem Cells

**DOI:** 10.1371/journal.pgen.1002054

**Published:** 2011-05-05

**Authors:** Grace X. Y. Zheng, Arvind Ravi, J. Mauro Calabrese, Lea A. Medeiros, Oktay Kirak, Lucas M. Dennis, Rudolf Jaenisch, Christopher B. Burge, Phillip A. Sharp

**Affiliations:** 1 MIT Koch Institute for Integrative Cancer Research, Cambridge, Massachusetts, United States of America; 2 Computational and Systems Biology Graduate Program, Massachusetts Institute of Technology, Cambridge, Massachusetts, United States of America; 3 Department of Biology, Massachusetts Institute of Technology, Cambridge, Massachusetts, United States of America; 4 Harvard-MIT Health Sciences and Technology Program, Cambridge, Massachusetts, United States of America; 5 Whitehead Institute for Biomedical Research, Cambridge, Massachusetts, United States of America; University of California San Francisco, United States of America

## Abstract

MicroRNAs (miRNAs) post-transcriptionally regulate the expression of thousands of distinct mRNAs. While some regulatory interactions help to maintain basal cellular functions, others are likely relevant in more specific settings, such as response to stress. Here we describe such a role for the mir-290-295 cluster, the dominant miRNA cluster in mouse embryonic stem cells (mESCs). Examination of a target list generated from bioinformatic prediction, as well as expression data following miRNA loss, revealed strong enrichment for apoptotic regulators, two of which we validated directly: Caspase 2, the most highly conserved mammalian caspase, and Ei24, a p53 transcriptional target. Consistent with these predictions, mESCs lacking miRNAs were more likely to initiate apoptosis following genotoxic exposure to gamma irradiation or doxorubicin. Knockdown of either candidate partially rescued this pro-apoptotic phenotype, as did transfection of members of the mir-290-295 cluster. These findings were recapitulated in a specific mir-290-295 deletion line, confirming that they reflect miRNA functions at physiological levels. In contrast to the basal regulatory roles previously identified, the pro-survival phenotype shown here may be most relevant to stressful gestations, where pro-oxidant metabolic states induce DNA damage. Similarly, this cluster may mediate chemotherapeutic resistance in a neoplastic context, making it a useful clinical target.

## Author Summary

In this study, we were interested in learning more about the roles of microRNAs—small segments of RNA that help turn off genes—during early development. By studying mouse embryonic stem cells, a unique cell type that can give rise to all adult tissues, we found that a number of genes related to cell survival appeared to be affected by global microRNA loss. Interestingly, these changes in gene expression did not lead to large increases in cell death during normal cell growth, but rather became apparent when cells were treated with agents that cause DNA damage, like the chemotherapeutic doxorubicin and gamma irradiation. Our results suggest that these microRNAs may provide robustness for mammalian development, ensuring proper development despite variations in blood flow and oxygen tension, known to cause DNA damage. Given that certain cancers share features of the embryonic state, including rapid proliferation and lack of differentiation, our results also suggest that the re-expression of these microRNAs in tumors may confer resistance to chemotherapeutic drugs.

## Introduction

MicroRNAs (miRNAs) are endogenous ∼22 nt RNAs that regulate gene expression post-transcriptionally. In animals, the ability of miRNAs to accomplish this regulation depends on complementarity between mature miRNA sequences and their mRNA targets. Most commonly, partial binding of miRNAs leads to destabilization of mRNA transcripts and/or inhibition of productive translation, and in rare cases perfect complementarity instead causes target cleavage. Both in vitro experiments and bioinformatics have shown that matches to positions 2–7 of the miRNA, referred to as the miRNA “seed,” are generally required for effective miRNA-directed mRNA downregulation [1], [2].

The roles of miRNAs in mouse embryonic stem cells (mESCs) have been of particular interest, as this knowledge may shed light on key aspects of mammalian development and generate useful insights into reprogramming and cancer, both of which recapitulate aspects of an ESC expression state [3], [4]. In addition, the survival of mESCs in the absence of Dicer (Dcr), the key RNase III enzyme that generates mature miRNAs, makes them a unique model system for dissecting miRNA function [5], [6]. Several large-scale sequencing datasets [7], [8], [9] have revealed that the mir-290-295 cluster constitutes the dominant miRNA population in mESCs, giving rise to about 50% of all reads in these cells (Table S1). Many of the miRNAs in this cluster share the hexamer seed ‘AAGUGC,’ which is also expressed at much lower levels by the mir-302 and mir-467 clusters, contributing less than 5% of total reads (Table S2). A similar percent contribution to total miRNA levels comes from the miR-17-92 family, which contains the shifted seed ‘AAAGUG,’ and therefore may share some common targets (Table S2) [7], [8], [9]. Given the abundance of the mir-290-295 cluster and these related sequences, much of mESC miRNA physiology is likely to be a function of this dominant seed sequence.

Within the mir-290-295 cluster, the ‘AAGUGC’ seed is found in miR-290-3p, miR-291a-3p, miR-291b-3p, miR-292-3p, miR-294, and miR-295. Consistent with their high expression, these miRNAs (which we shall refer to as the mir-295 cluster) have been linked to a number of functions in ES cells including maintenance of pluripotency and proliferation. For instance, miR-290-295 miRNAs have been shown to target Rbl2, which controls the expression of Dnmt3a and Dnmt3b [10], [11], suggesting a role for this miRNA cluster in regulating *de novo* DNA methylation. In addition, miR-290-295 miRNAs have been found to accelerate cell proliferation by promoting the G1 to S phase transition through targets such as p21 and Lats2 [12]. However, additional roles for this cluster remain to be elucidated.

Using a combination of target prediction data with microarrays of mESCs before (Dcr WT) and after (Dcr KO) miRNA loss, as well as before (295 WT) and after (295 KO) specific deletion of the mir-295 cluster (Medeiros *et al.*, manuscript in preparation), we have identified novel targets of the mir-295 cluster in ES cells. Initial analysis suggested strong enrichment of targets involved in apoptosis, a function that to date has not been linked to ES-cell specific miRNAs. Through gain and loss of function studies, we show that miR-290-295 miRNAs indeed serve a protective function in preventing mESC apoptosis during exposure to genotoxic stress. This protective effect appears to be mediated in part by direct repression of two novel targets, Caspase 2 and Ei24. As activation of these targets is dependent on DNA damage, we propose that their regulation may be particularly relevant during physiological stress in embryonic development. In addition, given prior indications that these two genes act as tumor suppressors, misexpression of this cluster in the context of cancer may promote resistance to standard genotoxic therapeutics.

## Results

### Predicted targets of the mir-295 cluster are enriched in pathways regulating apoptosis

**Figure 1:**
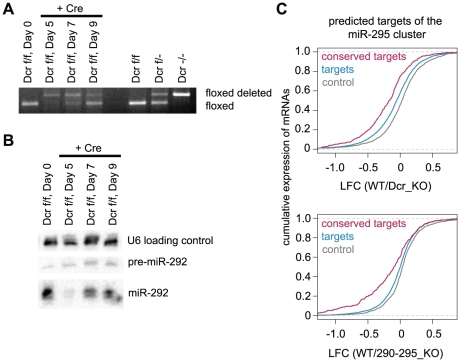
Acute Dicer deletion and specific mir-295 cluster deletion show global target derepression signatures. A. PCR for confirmation of Dicer loss on acute deletion. Dicer floxed and floxed-deleted bands were amplified over a nine day time course to determine the point of maximal enrichment for Dicer null cells. B. Northern blot for mature miRNAs following Dicer deletion. A DNA probe for the abundant miR-292 was assessed at 5, 7, and 9 days following Dicer deletion, with a probe for U6 to control for loading. C. Cumulative distribution functions (cdfs) of log_2_ fold change (LFC) in mRNA expression between Dcr WT and Dcr KO ES cells (top panel) and 295 WT and 295 KO ES cells (bottom panel) are plotted. Plots include *conserved targets* (red line), all predicted miR-295 *targets* (blue line), and *control* mRNAs (grey line). *targets* include ∼3000 predicted TargetScan targets of the miR-295 cluster. *Conserved targets* contains the ∼300 genes in the top 10% of *targets* ranked by TargetScan 5.1 branch length scores. The *control* mRNA set was selected to match the predicted targets in expression, 3' UTR length and composition. *Targets* are derepressed in both Dcr KO as well as 295 KO mESCs (*p*≤2.2e^−16^ and *p* = 9.7e^−9^ by rank sum test, respectively). Similarly, *conserved targets* are derepressed in both Dcr KO as well as 295 KO mESCs (*p*≤2.2e^−16^ and *p* = 3.1e^−14^ by rank sum test, respectively).

In order to identify additional endogenous targets of mESC miRNAs, we performed expression profiling of mESCs following Cre recombinase-mediated Dcr deletion using a previously characterized floxed Dcr mESC line [13], [14], [15]. As Dcr deletion leads to slower proliferation [6], [12], acute loss was examined in a polyclonal population, averaging over potential clonal variants and enriching for initial miRNA-mediated derepression rather than subsequent compensatory changes. Indeed, expression profiling from 3 biological replicates taken 5 days following deletion, a time point by which cells were predominantly Dcr null and a majority of miRNAs were lost (Figure 1A and 1B), showed better clustering than 3 chronic deletion cell lines, as indicated by higher Pearson correlation coefficients (Figure S1). To confirm that targets of the mir-295 cluster show a transcriptome-wide signature in this dataset, we calculated a cumulative density function (cdf) plot comparing expression differences for the set of all mir-295 cluster targets as determined by Targetscan 5.1 [16]. Relative to a control set of genes (*control*) matched for 3' UTR length, dinucleotide composition, and expression level, the mir-295 cluster target set (*targets*) was more derepressed upon Dcr loss (Figure 1C). An even larger derepression was seen for conserved mir-295 cluster targets (*conserved targets*), suggesting further enrichment of genuine targets in this set (Figure 1C). This observation supports the utility of these expression data for target discovery.

**Figure 2:**
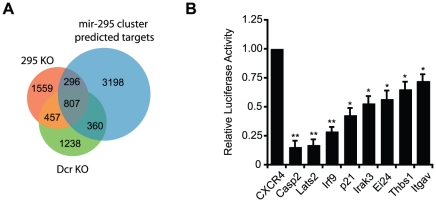
Microarray data and Targetscan predictions identify candidate miR-295 targets in mESCs. A. Venn diagram of microarray and target prediction data used to generate mir-295 cluster target candidates. “mir-295 cluster predicted targets”  =  predicted TargetScan targets of the miR-295 cluster; “295 KO”  =  genes that showed a 1.2 fold upregulation on mir-295 cluster loss; “Dcr KO”  =  genes that showed a 1.2 fold upregulation on Dicer loss. Only ES-expressed genes (i.e. genes with an expression of at least 16 on the wild type arrays) were considered for analysis. B. Activity of luciferase reporters of predicted targets of the mir-295 cluster were assayed in Dcr WT and Dcr KO ES cells. Luciferase reporters contain full length 3' UTRs of predicted targets. Relative luciferase activity is the ratio of the reporter’s activity in WT ES cells and Dcr KO ES cells.

To better understand the global effects of miRNA loss in ESCs, we next performed Gene Ontology (GO) analysis on an initial candidate set. Enrichment in specific GO categories was tested for all genes that increased on Dcr loss (defined as ≥1.2 fold up-regulation). The top statistically significant categories included “Regulators of Apoptosis” and “Cell Cycle” (*p* = 2.1e^−8^ and *p* = 5.6e^−5^, respectively). We further refined our candidate list using available array data from the 295 KO line, which also showed cdf plot signature changes for mir-295 cluster targets (Figure 1C) (Medeiros *et. al*., manuscript in preparation). In all, 807 candidates were identified as Targetscan-predicted targets of the cluster that showed at least a 1.2 fold up-regulation in knockout populations from both datasets (Figure 2A, Table S3). Over 40% of upregulated transcripts were shared between the Dcr KO and 295 KO lines, consistent with the finding that the mir-295 cluster contributes around half of all miRNAs in ES cells. The fact that this overlap is not even greater may be due to direct and indirect effects of non-AAGUGC seeds, as there is significantly more overlap – in fact, closer to 70% – between the two data sets when considering only those genes that are Targetscan-predicted AAGUGC targets (*p*<0.001, Fischer’s exact test).

Several candidate target genes were selected for further examination based on their degree of upregulation in Dcr KO and 295 KO cells, as well as their functional annotations. The tested targets span a range of biological functions and processes, from cell cycle regulators Lats2 and p21 to immunological signal transduction components Irf9 and Irak3. Their 3' UTRs were cloned into luciferase constructs, and expression levels between Dcr WT and Dcr KO cells were evaluated (Figure 2B). All candidates tested displayed at least mild repression relative to a control construct lacking miRNA target sites, ranging from strong (∼5-fold) to modest (∼30%) down-regulation. The magnitude of repression for the previously identified miR-295 targets Lats2 and p21 was comparable to that observed previously [12]. Additional transfection studies confirmed that repression could be conferred specifically by miR-295 in a Dcr KO background (Figure S2). These *in vitro* results support the enrichment of our candidate list for true miR-295 targets.

### Caspase 2 and Ei24, key apoptotic mediators, are direct targets of the mir-295 cluster

**Figure 3:**
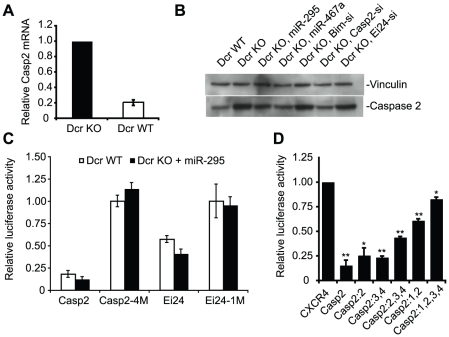
The pro-apoptotic genes Caspase 2 (Casp2) and Ei24 are direct targets of the mir-295 cluster. A. RT-PCR of Casp2 mRNA in Dcr KO cells. B. Western blot of Casp2 in Dcr WT and Dcr KO ES cells. 50 nM miR-295, miR-467a, Bim siRNA, Casp2 siRNA and Ei24 siRNA were transfected into Dcr KO ES cells, and Casp2 protein expression was assayed 24 hours later. miR-467a shares the same hexamer seed with miR-295, and Bim siRNA and Ei24 siRNA served as negative controls. C. Luciferase reporters with a full length Casp2 3' UTR or Ei24 3' UTR, as well as their seed mutant versions were assayed by comparing their activities in Dcr WT and Dcr KO ES cells. Casp2:4M has all 4 AAGUGC seed binding sites mutated, and Ei24:1M has its single AAGUGC seed binding site mutated. 20 nM miR-295 was transfected into Dcr KO ES cells and the relative activity measured versus non-transfected cells to test if the repression of luciferase reporters is specifically due to AAGUGC miRNAs. D. Casp2 luciferase reporters bearing different combinations of AAGUGC seed binding site mutations were tested in Dcr WT and Dcr KO ES cells. *Casp2:2*, 2nd AAGUGC binding site was mutated; *Casp2:3,4*, 3rd and 4th binding sites were mutated; *Casp2: 2,3,4*, 2nd, 3rd, and 4th binding sites were mutated; *Casp2:1,2*, 1st and 2nd binding sites were mutated; *Casp2: 1,2,3,4*, all binding sites were mutated. n≥3 for all experiments, and results are shown as mean ± S.E.M. (standard error of the mean). P-values were obtained by t-tests, * denotes *p* ≤ 0.05, and ** denotes *p* ≤ 0.01.

We chose to more closely examine one of the most strongly down-regulated reporter targets, Caspase 2 (Casp2), along with Ei24, as these targets could provide a novel link between ESC-specific microRNAs and cell survival. Casp2, an initiator of apoptosis in response to genotoxic stress [17], has four AAGUGC binding sites in its 3' UTR. Quantitative RT-PCR analysis demonstrated an approximately 5-fold increase in Casp2 transcript levels in Dcr KO cells, consistent with the degree of derepression observed with the luciferase reporter assay (Figure 3A). This observation indicates that the majority of miRNA repression likely occurs at the level of transcript stability. In support of the reporter assay, Dcr KO cells showed a comparable increase in Casp2 at the protein level, which could be partially rescued by transfection of either miR-295, miR-467a (which shares the same hexamer seed), or a Casp2 siRNA, but not by siRNAs against other unrelated targets (Figure 3B). Transfection of miR-295 also strongly repressed an intact Casp2 reporter in these cells, but not a reporter in which the four target sites were mutated (Figure 3C). Combinatorial mutagenesis revealed that repression was not conferred equally by these four sites, as much of the repression was lost by mutation of the first two sites alone (Figure 3D). Taken together, these data suggest that direct miRNA-mediated repression of Casp2 leads to approximately 5-fold repression, making it one of the most potently repressed mir-295 cluster targets identified to date.

We additionally characterized the novel target Ei24, which has also been implicated in apoptosis. Originally identified as a direct p53 transcriptional target that binds Bcl2 [18], [19], the Ei24 3' UTR contains one 7mer miR-295 site. The 3' UTR of Ei24 fused to a luciferase reporter conferred approximately 2-fold repression in Dcr WT cells relative to Dcr KO cells, an effect that could be restored following transfection of miR-295 (Figure 3C). Notably, repression was lost upon mutation of the seed site, confirming that Ei24 is a direct target.

### Mir-295 cluster miRNAs promote survival of ES cells during genotoxic stress

**Figure 4:**
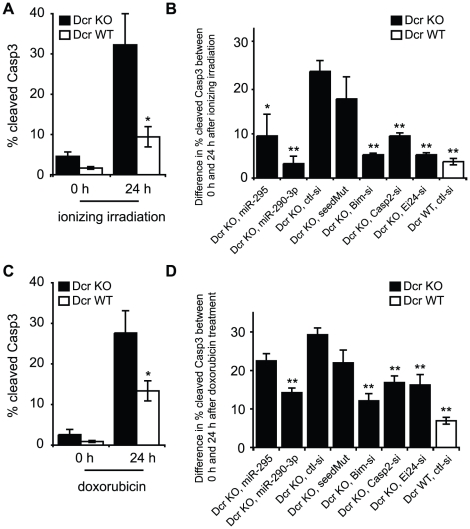
Downregulation of Casp2 and Ei24 partially rescues the increased apoptotic rate of Dcr KO cells following genotoxic stress. A. The percentage of cells expressing cleaved Caspase 3 (Casp3) in Dcr WT and Dcr KO ES cells after exposure to 5-Gy radiation (0 and 24 hours after radiation treatment). Cleaved Casp3 was assayed by flow cytometry, and was used to estimate apoptotic response. Apoptosis rate of Dcr KO cells is shown in black bars, and that of WT cells is shown in white bars. B. Dcr KO cells were treated with 5-Gy radiation 24 hours after transfection of 50 nM miR-295, miR-290-3p, Bim siRNA, Casp2 siRNA or Ei24 siRNA. Casp3 activity was assayed 0 and 24 h after the treatment, and the difference in apoptosis rate is shown. Transfection of seed mutants and control siRNAs into Dcr KO cells, and transfection of control siRNAs into WT cells served as controls. C,D. A similar series of experiments was performed in Dcr WT and Dcr KO cells using 100 nM doxorubicin. n≥3 for all experiments. Results are shown as mean ± S.E.M. P-values were obtained by Mann-Whitney tests, * denotes *p*≤0.05, and ** denotes *p*≤0.01.

Based on these repression data as well as the earlier informatic predictions, we tested whether mir-295 cluster miRNAs could modulate apoptosis in mESCs. The basal apoptosis rates of Dcr WT and KO ES cells in a 24 h period were compared by staining them with antibodies against cleaved Caspase 3 (Casp3) and then analyzing cells by flow cytometry. Under these conditions, only modest basal apoptotic rates were observed, with Dcr KO ES cells showing a slightly higher apoptosis rate than Dcr WT cells (Figure 4A, Figure S3A). Given that ESCs are highly sensitive to DNA damage [20] and both validated targets have been implicated in the DNA damage response, we hypothesized that the mir-295 cluster may be specifically protective in the context of genotoxic stress. To test this, we first examined the effect of exposing WT and Dcr KO cells to gamma irradiation or doxorubicin. Gamma irradiation induces DNA damage and activates ATM and p53, as does doxorubicin, a topoisomerase II inhibitor [21]. These signals lead to activation of the intrinsic apoptosis pathway and result in the cleavage of Casp3 [22]. We were able to confirm this cleavage product by Western blot in our cell culture system, as well as cleavage of Nanog, a previously reported Casp3 target [23] (Figure S5A). We also observed a decrease in Casp2 levels and the appearance of the previously described 35kD cleavage product [24], confirming its activation in our system (Figure S5B). Because this band was specific to DNA damage induction, the upregulation of Casp2 in Dcr KO cells appears to be insufficient to generate autocleavage. Both Dcr WT and KO ES cells showed minimal Casp3 activation immediately after 5-Gy gamma-irradiation, in line with previous descriptions of a 1–2 h lag phase in its activation (Figure 4A) [25]. However, there was a notable difference in their responses 24 h after the treatment (and to a lesser extent 10 h after treatment, Figure S4A); while 10% of WT cells became apoptotic, more than 30% of the Dcr KO cells exhibited Casp3 activity (Figure 4A). Similar results were seen using Annexin V staining, a complementary assay for detecting early apoptosis (Figure S3C). Importantly, mature miRNA levels from the mir-295 cluster were unchanged by these stressors (Figure S3D). Therefore, it appears that loss of mature miRNAs leads to an enhancement of apoptosis in the presence of DNA damage.

To examine whether the miR-295 targets modulated apoptosis, we transfected a series of siRNAs into Dcr KO cells and evaluated cell death following irradiation. The difference in Casp3 activation between 0 and 24 h timepoints was calculated in order to account for differences in transfection-specific toxicity (Figure S6A and S6B). Relative to control siRNAs, transfection of miR-290-3p or miR-295 drastically decreased the apoptosis response of Dcr KO cells to gamma irradiation (Figure 4B, Figure S6A and S6B). The reduction in apoptosis is specific to the AAGUGC seed, as seed mutants failed to rescue Dcr KO ES cells from apoptosis. When we applied siRNAs specific to each validated target, or to Bim, a well-characterized proapoptotic factor, cells exhibited a decrease of 5–10% in Casp3 activation 24 h after irradiation, a level similar to mir-295 cluster miRNA overexpression (Figure 4B, Figure S6A and S6B). Similar findings were obtained when cells were treated with 100 nM –300 nM doxorubicin, suggesting that the identified pathways are relevant to DNA damage in general (Figure 4C and 4D, Figure S4B, Figure S6C and S6D).

### Specific deletion of the mir-295 cluster enhances susceptibility to apoptosis upon DNA damage

**Figure 5:**
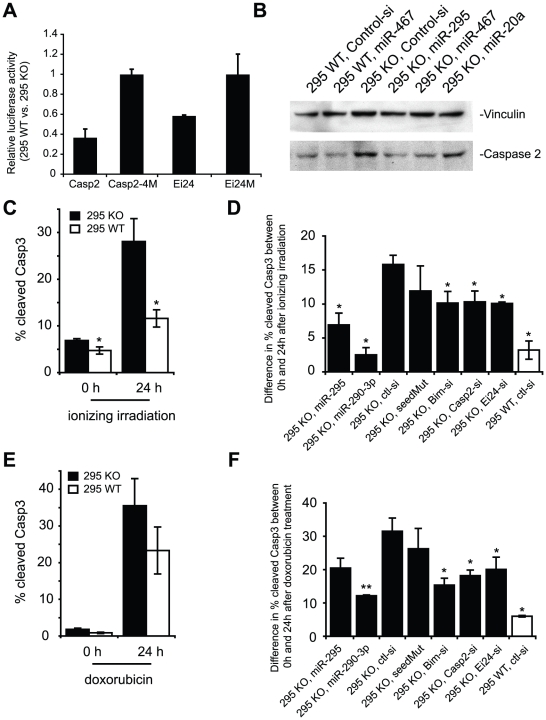
Loss of the mir-295 cluster derepresses Casp 2 and Ei24 3' UTRs and enhances sensitivity to DNA damaging agents. A. Luciferase reporters with full length versions of the Casp2 3' UTR or Ei24 3' UTR, as well as their seed mutant versions were assayed in 295 WT and 295 KO ES cells. B. Western blot of Casp2 in 295 WT and 295 KO ES cells. 50 nM miR-295, miR-467a, and miR-20a were transfected into 295 KO ES cells, and Casp2 protein expression was assayed 24 hours after the transfection. C. The percentage of cells with cleaved Casp3 in 295 WT and 295 KO ES cells after exposure to 5-Gy radiation (0 and 24 hours after radiation treatment). Cleaved Casp3 was assayed by flow cytometry, and was used to estimate apoptotic response. Apoptosis of 295 KO cells is shown in black bars, and that of 295 WT cells is shown in white bars. D. Cells were transfected with 50 nM siRNAs as shown, and difference in apoptotic response of 295 WT and 295 KO ES cells 24 hours after exposure to 5-Gy radiation was plotted. E,F. A similar series of experiments was performed on 295 WT and 295 KO cells after exposure to 100 nM doxorubicin. n≥3 for all experiments. Results are shown as mean ± S.E.M. (standard error of the mean). P-values were results of Mann-Whitney tests, and * denotes *p*≤0.05, and ** denotes *p* ≤ 0.01.

Because deletion of Dcr involves global miRNA loss, and three additional clusters containing the same or similar hexamer seed, mir-302, mir-467, and mir-17-92, are expressed in ESCs (Table S2), we examined the 295 KO line to determine the specific contribution of the mir-295 cluster to cell survival. Genetic deletion offers the best insight into physiological function as it avoids overexpression artifacts of exogenous miRNAs or toxicity effects of miRNA inhibitors. This system also avoids confounding by alternative miRNA-independent roles for Dcr itself in cell survival, as have been recently reported in *C. elegans* [26]. We first re-examined the reporter constructs for Casp2 and Ei24 in the 295 KO ESC line relative to its wild-type counterpart. In this context, the Casp2 reporter was derepressed approximately half as strongly as it was in Dcr KO cells, suggesting that the miR-302 and miR-467a families of miRNAs incompletely compensate for loss of the mir-295 cluster (Figure 5A). This partial derepression in the mir-295 cluster deletion probably reflects the quantitative change in the total level of AAGUGC seed miRNAs, as exogenous miR-295 could further repress Casp2 protein levels (Figure 5B). We next exploited 295 KO ES cells to determine whether these cells had an increase in apoptosis upon exposure to DNA damaging agents. 295 WT and KO ES cells were irradiated and the level of cleaved Casp3 activity was measured 0 and 24 h after treatment (Figure 5C, Figure S3B). As expected, 295 KO cells were much more sensitive to irradiation than their WT counterparts (Figure 5C). Again, overexpression of two miRNAs in the cluster, miR-290-3p and miR-295, reduced the rate of apoptosis (Figure 5D, Figure S7A and S7B). In addition, knockdown of the validated targets Casp2 or Ei24, or the pathway component Bim, partially rescued cells from apoptosis caused by irradiation (Figure 5D, Figure S7A and S7B). We repeated these experiments with 100 nM doxorubicin as before, obtaining similar results (Figure 5E and 5F, Figure S7C and S7D). Therefore, deletion and restoration of mir-295 cluster miRNAs recapitulate the modulation of apoptosis rates seen in a Dcr null context.

## Discussion

Here, we provide the first demonstration that the mir-295 cluster can suppress apoptosis in mESCs following exposure to the genotoxic stressors ionizing irradiation and doxorubicin. Initially suggested by an informatic comparison of global expression data following Dicer loss, the link between ESC miRNAs and cell death may act in part through the novel targets Casp2 and Ei24. In the case of Casp2, this appears to occur through multiple seed match sites in the 3' UTR leading to a roughly 5 fold reduction in expression, while for Ei24, targeting is achieved through just a single complementary site conferring approximately a 2 fold repression.

Although the exact functions of these two mediators are still emerging, multiple lines of evidence suggest that they are important in cell survival. Initial studies of Casp2 knockout mice showed increased numbers of oocytes suggesting resistance to cell death, which was confirmed by their decreased sensitivity to doxorubicin [27]. Subsequent studies have extended this pro-survival phenotype of Casp2 loss to include a number of tissues and DNA damaging agents [28]. Ei24, which was originally identified in a screen for etoposide-induced transcripts, has been shown to promote cell death by binding and sequestering Bcl2 [19].

**Figure 6:**
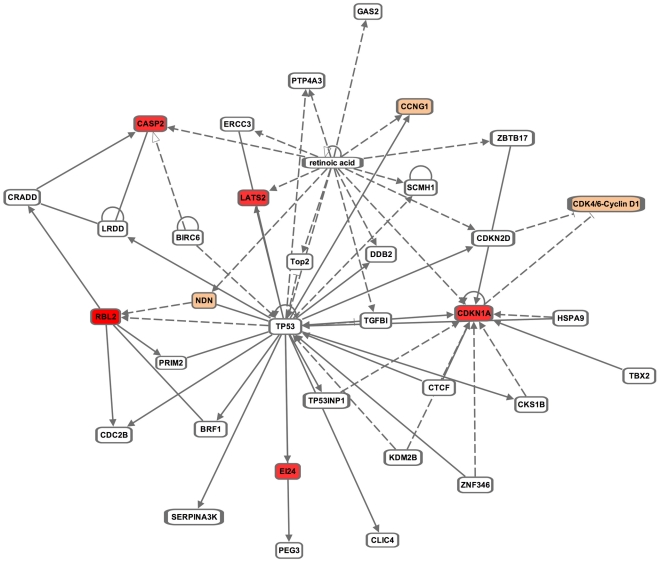
Ingenuity Pathway Analysis (IPA) of miR-295 targets. IPA was performed for validated targets of miR-295 from this and prior studies [10], [11], [12], and identified the network, “Cell Death, Cell Cycle, Cellular Function and Maintenance,” which centers around p53. Solid lines indicate direct interactions and dashed lines indicate indirect interactions. Validated targets of miR-295 are shown in red and computationally predicted targets are shown in orange.

Interestingly, these genes as well as several previously identified miR-295 family targets are known to be directly or indirectly associated with p53. Indeed, Pathway Analysis of well-characterized miR-295 targets brought up a single significant network (*p* = 10^−14^), “Cell Death, Cell Cycle, Cellular Function and Maintenance,” which prominently featured p53 (Figure 6). Activation of Casp2 can occur through a protein complex in which it associates with the p53 target PIDD [28]. Ei24 itself is a p53 transcriptional target, identified as one of 14 genes induced by adenoviral transfection of p53 into a p53-null colon cancer cell line [19]. We additionally confirmed two previously identified miR-295 targets, p21 (also known as Cdkn1a) and Lats2 [29]. In the case of p21, direct activation by p53 promotes cell cycle arrest at the G1/S phase [30]. Lats2, while also induced by p53, exists in a positive feedback loop with p53 in which it binds and inhibits Mdm2, thereby activating p53 [31]. Thus, miR-295 family miRNAs target a number of p53 associated genes and in all cases antagonizing p53 activation, consistent with the protective effect we have identified here.

Like p53, the mir-295 cluster affects both arms of cellular proliferation, namely cell death and cell cycle progression [12], [29]. Unlike cell cycle progression, however, the anti-apoptotic role is likely to have the greatest developmental consequences following DNA damage, induced physiologically by oxidative stress or metabolites. Interestingly, even simply *ex vivo* cell passage may be sufficient to induce a low level of stress, as evidenced by the slightly higher apoptotic rate of Dicer null cells under basal culture. In their pro-survival capacity, these miRNAs may confer robustness during embryonic development, as has been demonstrated in *Drosophila*. For instance, miR-7 has been shown to participate in a complex network of feedback loops to ensure proper photoreceptor cell development despite temperature fluctuations in development [32]. Similarly, miR-263a/b appear to prevent patterning defects in bristle formation, again consistent with the notion that they promote the fidelity of developmental trajectories [32], [33]. Early phenotypic data from mir-290-295 KO mice suggests an incompletely penetrant gestational phenotype (Medeiros *et. al*., manuscript in preparation), supporting the model that loss of this cluster is tolerated in certain developmental scenarios, perhaps including those with limited stressors during gestation.

Beyond regulating development, the miRNAs described here may also have important consequences for cancer, as both Casp2 and Ei24 are considered tumor suppressors. In the case of Casp2, this has been best demonstrated in the Eu-myc lymphoma model, where loss of even a single copy of Casp2 can accelerate malignant transformation [34]. Similarly, Ei24 is found in a region that shows frequent loss-of-heterozygosity in solid tumors, and its loss has been associated with increased breast cancer invasiveness [19]. In addition, knockdown of Ei24 in mouse fibroblasts or human breast cancer cell lines leads to increased resistance against etoposide-induced apoptosis [19], [35]. Consistent with these findings, the mir-295 cluster itself has been speculated to be an “oncomir” cluster, as overexpression of its human homolog, the mir-371-373 cluster, has been found in various human tumors [36], [37], [38] and may promote malignant transformation [39]. Given our findings, we hypothesize that these miRNAs may have a survival promoting function with dual effects, helping cells navigate physiological stresses during development, and helping cancer cells maintain viability in the face of genotoxic chemotherapeutic agents.

In conclusion, these data expand our understanding of ESC miRNA function, linking the ES cell specific miR-295 family to key players in cell death. Further, this analysis reveals a complex relationship between embryonic stem cell miRNA regulation and p53 activation.

## Materials and Methods

### ES cell culture

Feeder-free Dicer1^flox/flox^ and Dicer1^−/−^ mouse embryonic stem cells (mESCs) were generated and maintained on gelatin as described previously [40]. mESCs cells containing a floxed and excised mir-295 cluster were generated in a similar manner and will be described in an upcoming publication (Medeiros *et. al*., manuscript in preparation).

### Oligos and siRNAs used in all the experiments

See Table S4.

### Generation of luciferase constructs, mESC transfection, and luciferase assays

MicroRNA mediated repression of each candidate gene was tested by cloning PCR amplified products corresponding to the entire 3' UTR downstream of a pRL-CMV Renilla luciferase reporter as described previously [41]. Nucleotides 5–7 of Casp2, Bim, and Ei24 binding sites were mutated by Quickchange site-directed mutagenesis. Digests were performed using either XhoI or SalI to give the 5' site and ApaI or NotI to give the 3' site. Firefly luciferase (pGL3) was used as a transfection control. Data shown are summaries of three or more independent trials. 24 hours before transfection 1e^5^ mESC cells were plated/well of gelatinized 24-well plate. Cells were transfected with 2 µl Lipofectamine 2000 (Invitrogen), 0.1 µg of CMV-GFP plasmid (Invitrogen), 0.7 µg of pWS (carrier plasmid), and 50 nM siRNAs in 300 µl of Opti-MEM (Invitrogen). 4 hours after transfection, transfection mix was removed from cells and replaced with ESC media. 24 hours after transfection, cells were lysed with 1X Passive Lysis Buffer (Promega) and Dual luciferase was measured using Dual Luciferase reporter assay system (Promega) according to manufacturer’s instructions.

### Northern Blot analysis

Total RNA was isolated from ES cells with or without acute Dicer deletion using Trizol (Invitrogen), following the standard protocol. Approximately 50 µg of each RNA was loaded onto a 15% denaturing MOPS gel, according to the Northern Blot protocol outlined previously [42]. Membranes were probed for miR-292 and exposed to a phosphoimager before scanning. Prior to hybridizing with a different probe, membranes were stripped by incubating the membrane in boiling 0.1% SDS for 30 minutes and loss of signal was confirmed prior to rehybridization.

### Western Blot analysis

24 hours after transfection with short RNAs, Dicer1^−/−^, Dicer^flox/flox^, miR-290-295^−/−^, or miR-290-295^flox/flox^ cells were lysed in RIPA buffer (1% NP40, 0.5% sodium deoxycholate, 0.1% SDS, in pH 7.4 PBS) containing protease inhibitors. 30–50 µg lysate was loaded onto 8–12% Bis-Tris gels (Invitrogen) and wet-transferred at 4°C to Westran PVDF membranes for 2 h at 70V. After 1 h blocking at room temperature in 5% milk-TBST, membranes were probed overnight at 4°C with 1∶2000 mouse anti-vinculin (Santa Cruz Biotechnology) or 1∶200 rat anti-Caspase 2 (Millipore, 10C6). After 2×10 min. TBST washes, membranes were probed for 1 h at room temperature with 1∶2000 corresponding hRP-conjugated secondary, washed an additional 2×10 min. in TBST, and visualized using Western Lightning Plus ECL (PerkinElmer).

### RT-PCR

Trizol (Qiagen) was used to extract RNA from Dicer1^flox/flox^ and Dicer1^−/−^ cells. A Superscript III kit (Invitrogen) was used to reverse transcribe 1 µg RNA following DNAse treatment with the Turbo-DNA free kit (Ambion), and real time PCR was performed with the primer sequences listed, using beta actin for normalization.

### Transfection and Casp3 assays

24 hours before transfection 2e^5^ mESC cells were plated/well of gelatinized 12-well plates. Cells were transfected with 4 µl Lipofectamine 2000 (Invitrogen), 0.2 µg pCAGGS-mCherry plasmid, 1.4 µg of pWS, and 50 nM of siRNA in 600 µl of Opti-MEM (Invitrogen). 4 hours after transfection, transfection mix was removed from cells and replaced with ESC media. 24 hours after transfection, cells were exposed to 5-Gy gamma radiation or 100 nM doxorubicin. Immediately after exposure, one plate of cells were trypsinized and fixed with 1× BD Perm buffer. Cells were stained with Rabbit Anti-Casp3 antibody (BD Biosciences) at 1∶100 for 20 min. at room temperature. Following washing, cells were incubated with Alexa-488-conjugated secondary antibody (diluted 1∶250) (Invitrogen) for 60 min. at room temperature, washed, and resuspended in BD FACS buffer containing 1∶5000 Hoechst stain. 24 hours after the treatment, another plate of cells was trypsinized and treated with the same protocol for FACS analysis.

Casp3 assays were also performed on Dcr KO and WT mESCs without transfection. 24 hours before collecting cells for the 0 h time point for Casp3 assay, 2e^5^ mESC were plated/well of gelatinized 6-well plates. In the context of genotoxic stress, 4e^5^ mESCs were plated/well of gelatinized 6-well plates. 24 hours after plating, cells were treated with 5-Gy radiation or 100 nM doxorubicin. Casp3 assays were performed at 0 h and 24 h after the treatment following the same protocol described above.

### Annexin V assays

4e^5^ mESCs were plated/well of gelatinized 6-well plates. 24 h after plating, cells were exposed to 100 nM doxorubicin. Cells were trypsinized 0 h and 24 h after the treatment for Annexin V detection, following Annexin V-FITC apoptosis detection kit (BD Biosciences).

### Microarray analysis

Microarray analysis was performed 5 days following transfection of Dicer^flox/flox^ wild-type cells with either GFP alone or GFP and Cre recombinase, and data were analyzed using biological triplicates. Microarrays for the mir-295 cluster deletion were performed on two deletion and two wild-type lines independently derived. Spot replicates were condensed using geometric means.

The log fold change (LFC) value for Dcr WT/Dcr KO was defined as the difference between the mean log expression in Dcr WT cells and the mean log expression in Dcr KO cells. The conserved set of targets were downloaded from TargetScanMouse5.1 website (http://www.targetscan.org/mmu_50/). To identify targets predicted for the AAGUGC seed family, we looked at all miRNAs that contain AAGUGC in their seed region. More specifically, they include “miR-291b-3p/519a/519b-3p/519c-3p”, “miR-290-3p/292-3p/467a”, “miR-467cd”, “miR-106/302”, and “miR-467b”. We excluded all the targets of “miR-302ac/520f”, as well as T1A 7mer targets of “miR-467b”, as they do not contain the 6-mer match to AAGUGC. Targets with top 10% of branch length scores were considered “conserved”.

### Gene Ontology and Pathway analysis

Gene Set Analysis Toolkit (http://bioinfo.vanderbilt.edu/webgestalt/) was used to perform GO analysis. Targets and controls were generated as described in the text. Network data were analyzed through the use of Ingenuity Pathways Analysis (Ingenuity Systems, www.ingenuity.com). Ingenuity Pathway Analysis (IPA) was performed on the set of validated miR-295 targets to identify the most strongly associated canonical pathways.

### Statistical analyses

All test statistics were calculated using R (http://www.r-project.org). The Wilcoxon rank sum test was used because it does not assume normality of the underlying distributions. T-tests and Kolmogorov–Smirnov (KS) test using these data gave generally similar results.

## Supporting Information

Figure S1 Microarray data from polyclonal acute dicer deletion samples show better inter-sample correlation than data from clonal chronic dicer deletion lines. Biological triplicates were obtained for both 5 days post Dicer deletion (Acute 1-3) and over 1 month following Dicer deletion (Chronic 1-3), after which samples were normalized together. Pearson correlations for all pairwise comparisons within the two conditions are shown here.
(PDF)

Figure S2 Repression of predicted targets of the miR-295 cluster in Dcr WT and Dcr KO ES cells. Activity of luciferase reporters of predicted targets were assayed in WT, Dcr KO ES cells, as well as in Dcr KO ES cells after over expression of 20 nM miR-295. n≥2, and results are shown as mean ± S.E.M.
(PDF)

Figure S3 Comparison of WT and KO cells’ apoptosis response to stress. A. The percentage of cells expressing cleaved Caspase 3 (Casp3) in WT and Dcr KO ES cells under normal culturing conditions (0 and 24 hours after plating). B. The percentage of cells expressing cleaved Caspase 3 (Casp3) in 295 WT and 295 KO ES cells under normal culturing conditions (0 and 24 hours after plating). Cleaved Casp3 was assayed by flow cytometry, and was used to estimate apoptosis response. Apoptosis of KO cells is shown in black bars, and that of WT cells is shown in white bars. C. Annexin V positive cells were assayed by flow cytometry immediately or 24 h after exposure to 100 nM doxorubicin. n = 2, and results are shown as mean ± S.E.M. (standard error of the mean). D. Northern analysis for miR-295 in Dcr KO ES cells, WT ES cells, and WT ES cells 6 hours after 2 μM doxorubicin treatment.
(PDF)

Figure S4 Time course of WT and KO cells’ apoptosis response to stress. A. WT and Dcr KO ES cells were treated with 5-Gy radiation. Casp3 activity was assayed 0 h, 10 h, and 24 h after the treatment, and the differences in apoptosis rate (between 10 h and 0 h, and between 24 h and 0 h) are shown. Cleaved Casp3 was assayed by flow cytometry, and was used to estimate apoptosis response. Apoptosis rate of Dcr KO cells is shown in black bars, and that of WT cells is shown in white bars. n = 2, and results are shown as mean ± S.E.M. B. WT and Dcr KO cells were treated with 2 doses of doxorubicin (200 nM and 300 nM). Casp3 activity was assayed 0 h, 10 h, and 24 h after the treatment, and the differences in apoptosis rate (between 10 h and 0 h, and between 24 h and 0 h) are shown. n = 1 with 3 technical replicates, and results are shown as mean ± standard deviation.
(PDF)

Figure S5 Analysis of Caspase 2 and Caspase 3 cleavage upon DNA damage induction. A. Dcr KO cells treated with 2 μM doxorubicin for 6 hours. Blot was probed with an antibody specific for cleaved Caspase 3 and for its target, Nanog, before and after treatment. B. Dcr KO and Dcr WT cells following 2 μM doxorubicin treatment for 6 hours. Intact Caspase 2 (49 kD) and cleaved Caspase 2 (35 kD) are both shown.
(PDF)

Figure S6 Raw data of Dcr KO and WT cells’ apoptosis response after treatment of 5-Gy radiation or 100 nM doxorubicin. A. Dcr KO cells were treated with 5-Gy radiation 24 hours after transfection of 50 nM miR-295 or miR-290-3p. Caspase 3 activity was assayed 0 and 24 h after the treatment. Transfection of seed mutants and control siRNAs (50 nM) into Dcr KO cells, and overexpression of control siRNAs (50 nM) into WT cells served as controls. B. Dcr KO cells were treated with 5-Gy radiation 24 hours after transfection of 50 nM siRNAs against Bim, Casp2, and Ei24. Caspase-3 activity was assayed 0 and 24 h after the treatment. C,D. A similar series of experiments was performed in Dcr WT and Dcr KO cells using 100 nM doxorubicin. n ≥ 3 for all experiments. Results are shown as mean ± S.E.M. (standard error of the mean).
(PDF)

Figure S7 Raw data of 295 KO and 295 WT cells’ apoptosis response after treatment of 5-Gy radiation or 100 nM doxorubicin. A. 295 KO cells were treated with 5-Gy radiation 24 hours after transfection of 50 nM of miR-295 or miR-290-3p. Caspase 3 activity was assayed 0 and 24 h after the treatment. Transfection of seed mutants and control siRNAs (50 nM) into 295 KO cells, and overexpression of control siRNAs (50 nM) into 295 WT cells served as controls. B. 295 KO cells were treated with 5-Gy radiation 24 hours after transfection of 50 nM siRNAs against Bim, Casp2, and Ei24. Caspase-3 activity was assayed 0 and 24 h after the treatment. C,D. A similar series of experiments was performed in 295 WT and 295 KO cells using 100 nM doxorubicin. n ≥3 for all experiments. Results are shown as mean ± S.E.M. (standard error of the mean).
(PDF)

Table S1 Sequences and expression level of the mir-295 cluster in ES cells. The 6-mer seed is highlighted in bold. The cloning statistics were taken from previously published studies [7], [8], [9].
(PDF)

Table S2 Sequences and expression level of the mir-302, mir-467, and mir-17-92 clusters in ES cells. The 6-mer seed is highlighted in bold. The cloning statistics were taken from previously published studies [7], [8], [9].
(PDF)

Table S3 Predicted targets of the mir-295 cluster.
(PDF)

Table S4 Oligos and siRNAs used in all experiments.
(PDF)
