# Supplementary figures and images for: A Latent Pro-Survival Function for the Mir-290-295 Cluster in Mouse Embryonic Stem Cells
Source: PLoS Genet. 2011 May 5;7(5):e1002054. doi: 10.1371/journal.pgen.1002054 (PMC3088722; doi:10.1371/journal.pgen.1002054)

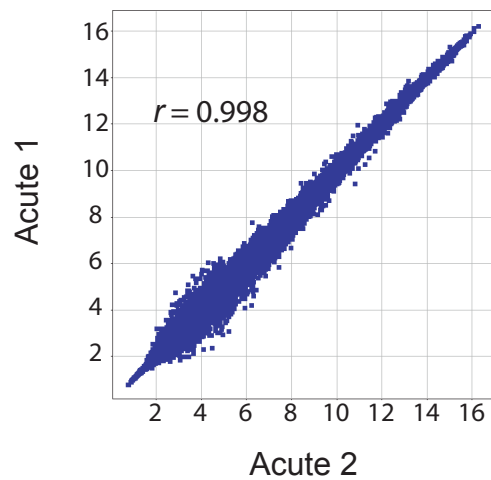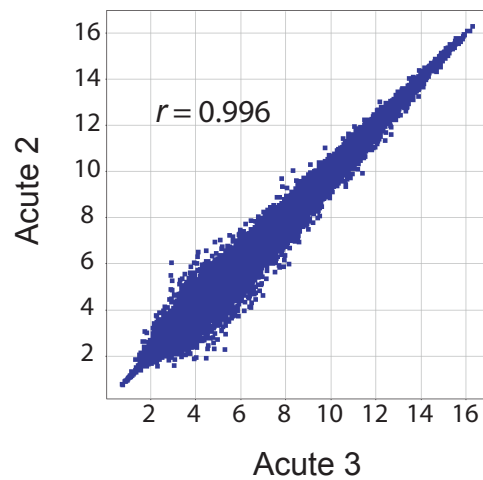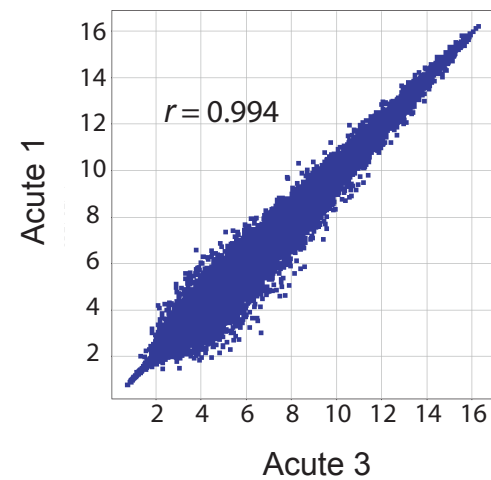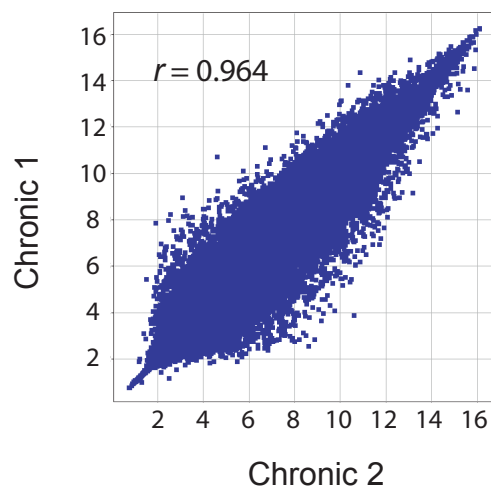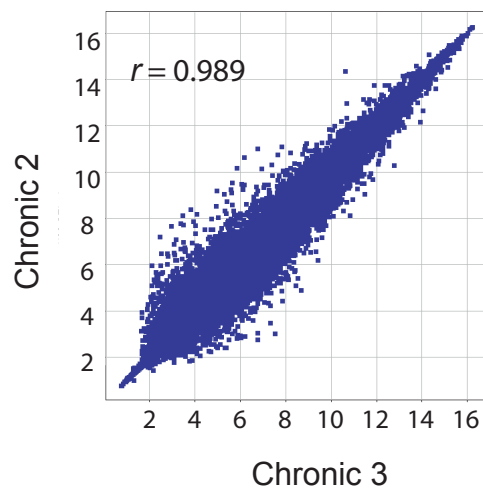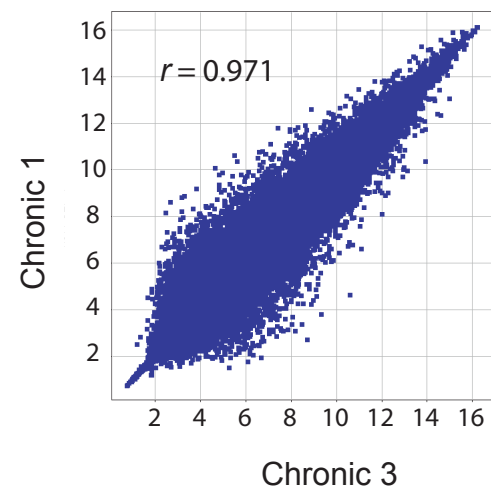

log2 expression

log2 expression

Supplement: Figure S1 — Microarray data from polyclonal acute dicer deletion samples show better inter-sample correlation than data from clonal chronic dicer deletion lines. Biological triplicates were obtained for both 5 days post Dicer deletion (Acute 1-3) and over 1 month following Dicer deletion (Chronic 1-3), after which samples were normalized together. Pearson correlations for all pairwise comparisons within the two conditions are shown here. (PDF) [file pgen.1002054.s001.pdf]

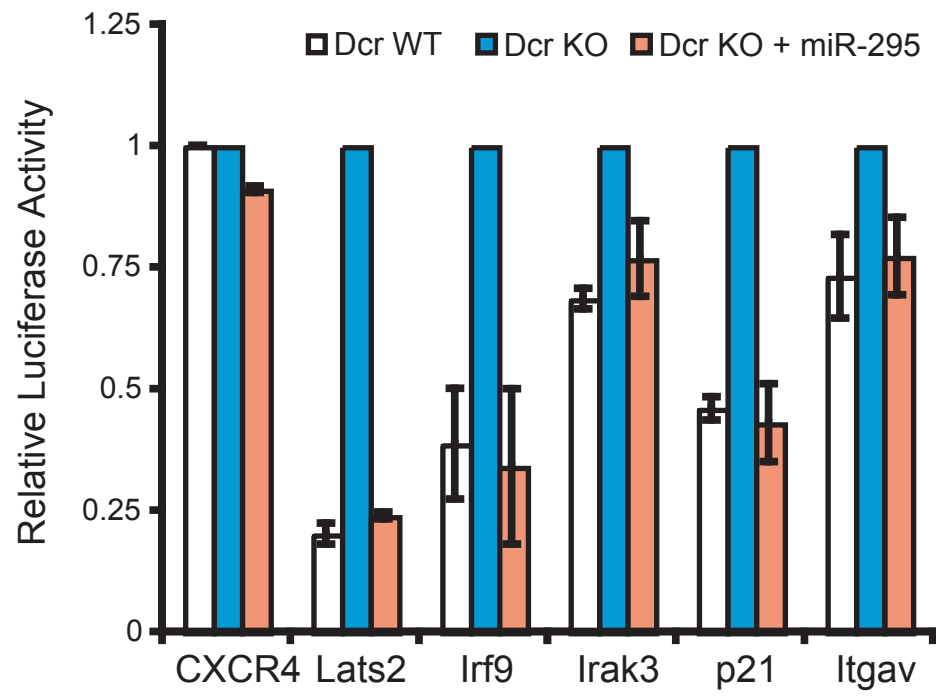

Supplement: Figure S2 — Repression of predicted targets of the miR-295 cluster in Dcr WT and Dcr KO ES cells. Activity of luciferase reporters of predicted targets were assayed in WT, Dcr KO ES cells, as well as in Dcr KO ES cells after over expression of 20 nM miR-295. n≥2, and results are shown as mean ± S.E.M. (PDF) [file pgen.1002054.s002.pdf]

**A**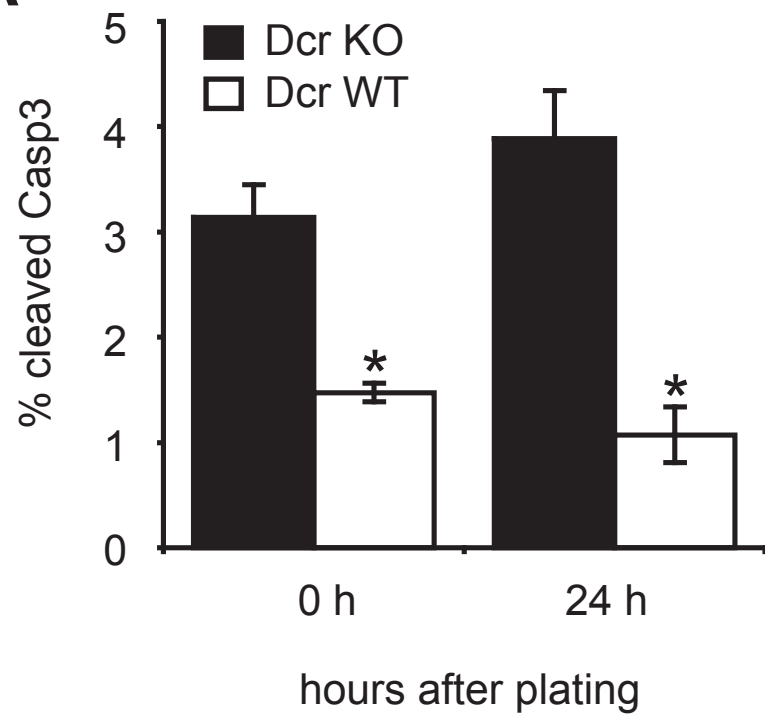**B**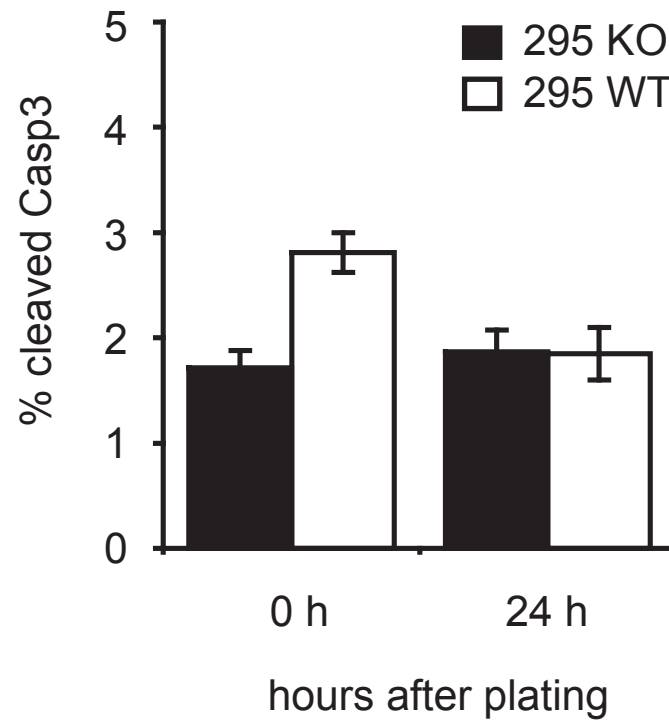**C**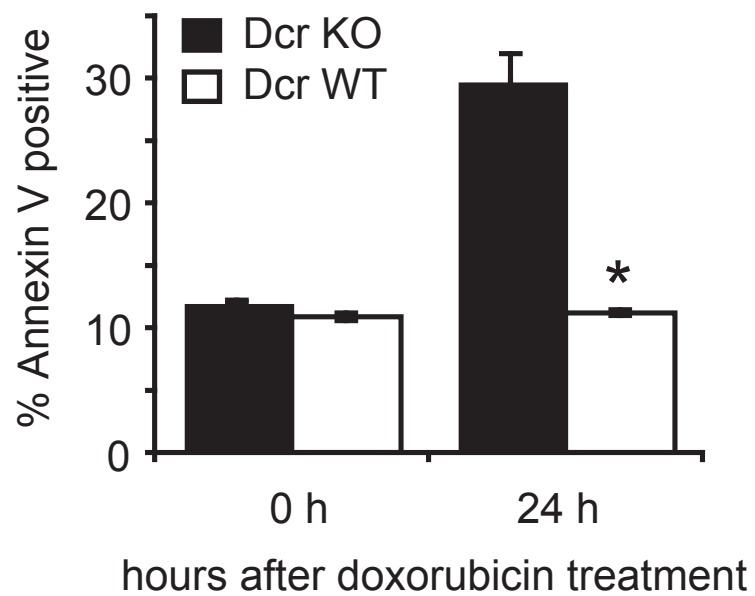**D**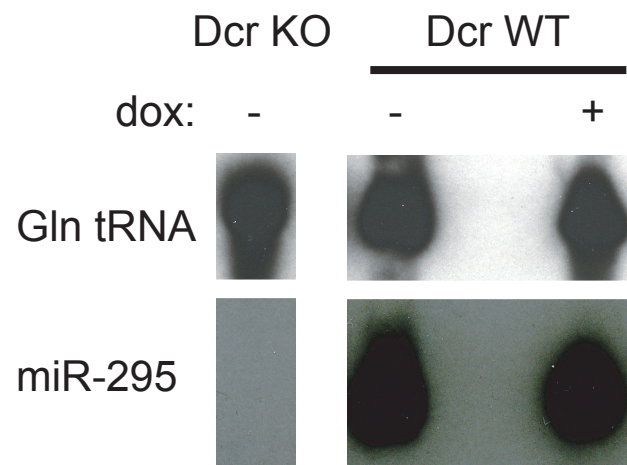

Supplement: Figure S3 — Comparison of WT and KO cells’ apoptosis response to stress. A. The percentage of cells expressing cleaved Caspase 3 (Casp3) in WT and Dcr KO ES cells under normal culturing conditions (0 and 24 hours after plating). B. The percentage of cells expressing cleaved Caspase 3 (Casp3) in 295 WT and 295 KO ES cells under normal culturing conditions (0 and 24 hours after plating). Cleaved Casp3 was assayed by flow cytometry, and was used to estimate apoptosis response. Apoptosis of KO cells is shown in black bars, and that of WT cells is shown in white bars. C. Annexin V positive cells were assayed by flow cytometry immediately or 24 h after exposure to 100 nM doxorubicin. n = 2, and results are shown as mean ± S.E.M. (standard error of the mean). D. Northern analysis for miR-295 in Dcr KO ES cells, WT ES cells, and WT ES cells 6 hours after 2 μM doxorubicin treatment. (PDF) [file pgen.1002054.s003.pdf]

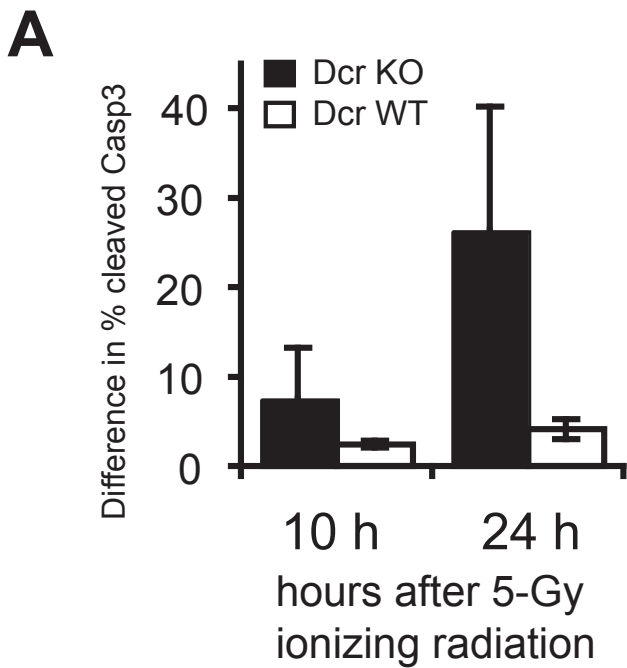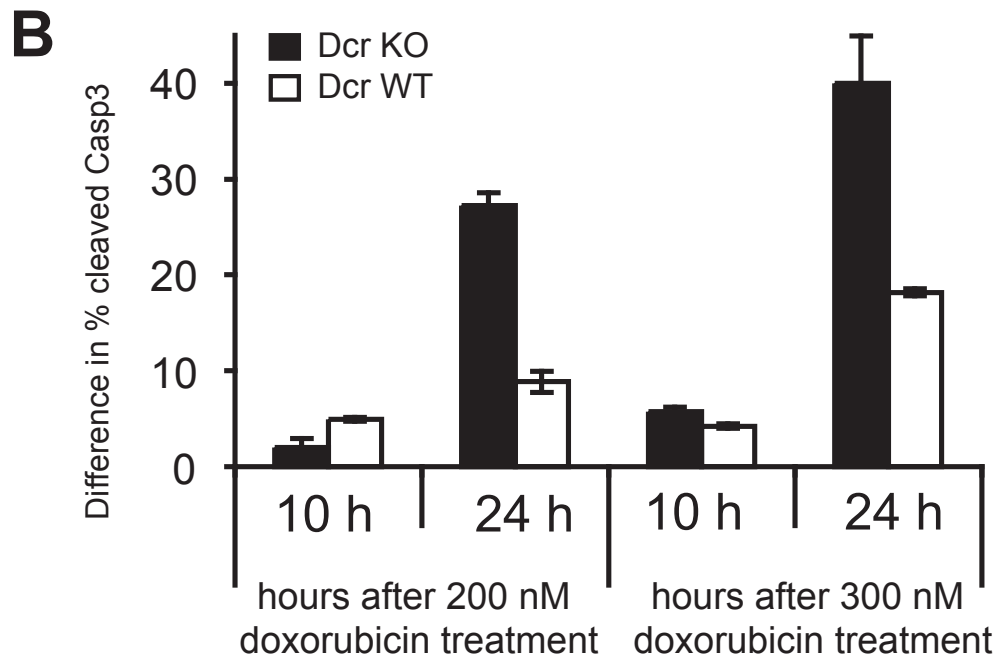

Supplement: Figure S4 — Time course of WT and KO cells’ apoptosis response to stress. A. WT and Dcr KO ES cells were treated with 5-Gy radiation. Casp3 activity was assayed 0 h, 10 h, and 24 h after the treatment, and the differences in apoptosis rate (between 10 h and 0 h, and between 24 h and 0 h) are shown. Cleaved Casp3 was assayed by flow cytometry, and was used to estimate apoptosis response. Apoptosis rate of Dcr KO cells is shown in black bars, and that of WT cells is shown in white bars. n = 2, and results are shown as mean ± S.E.M. B. WT and Dcr KO cells were treated with 2 doses of doxorubicin (200 nM and 300 nM). Casp3 activity was assayed 0 h, 10 h, and 24 h after the treatment, and the differences in apoptosis rate (between 10 h and 0 h, and between 24 h and 0 h) are shown. n = 1 with 3 technical replicates, and results are shown as mean ± standard deviation. (PDF) [file pgen.1002054.s004.pdf]

**A**

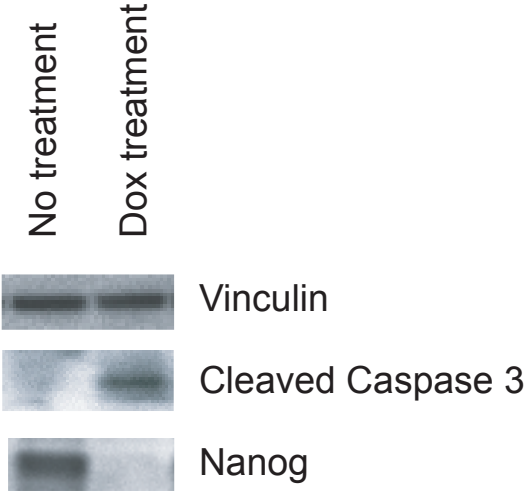

**B**

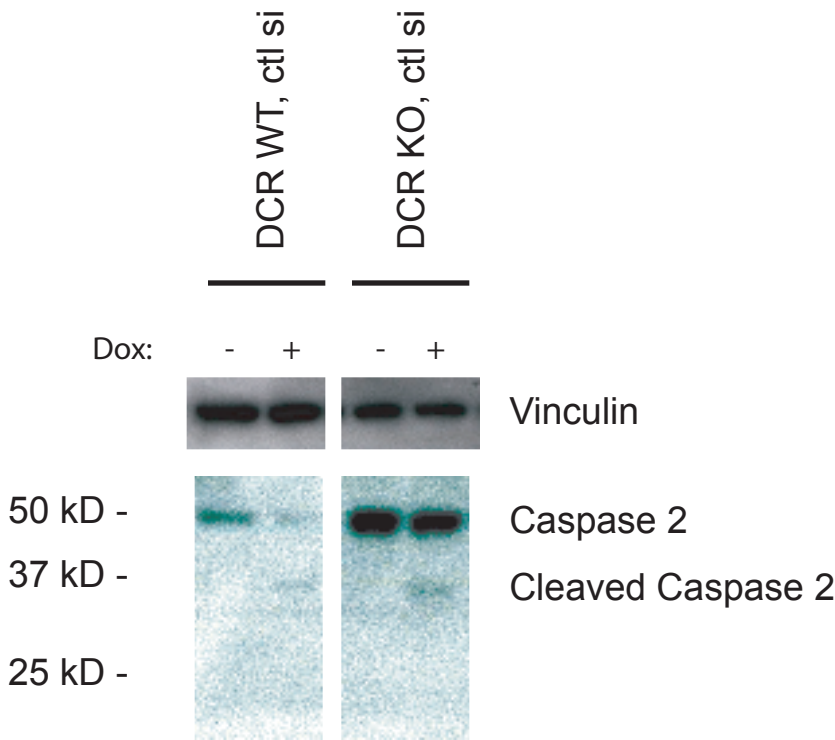

Supplement: Figure S5 — Analysis of Caspase 2 and Caspase 3 cleavage upon DNA damage induction. A. Dcr KO cells treated with 2 μM doxorubicin for 6 hours. Blot was probed with an antibody specific for cleaved Caspase 3 and for its target, Nanog, before and after treatment. B. Dcr KO and Dcr WT cells following 2 μM doxorubicin treatment for 6 hours. Intact Caspase 2 (49 kD) and cleaved Caspase 2 (35 kD) are both shown. (PDF) [file pgen.1002054.s005.pdf]

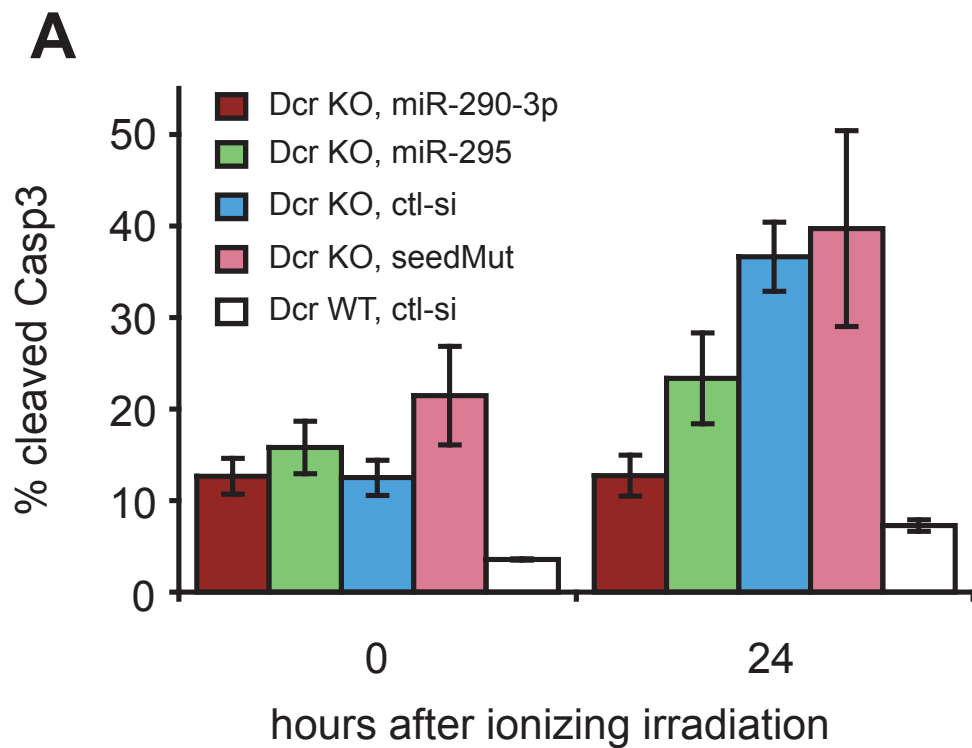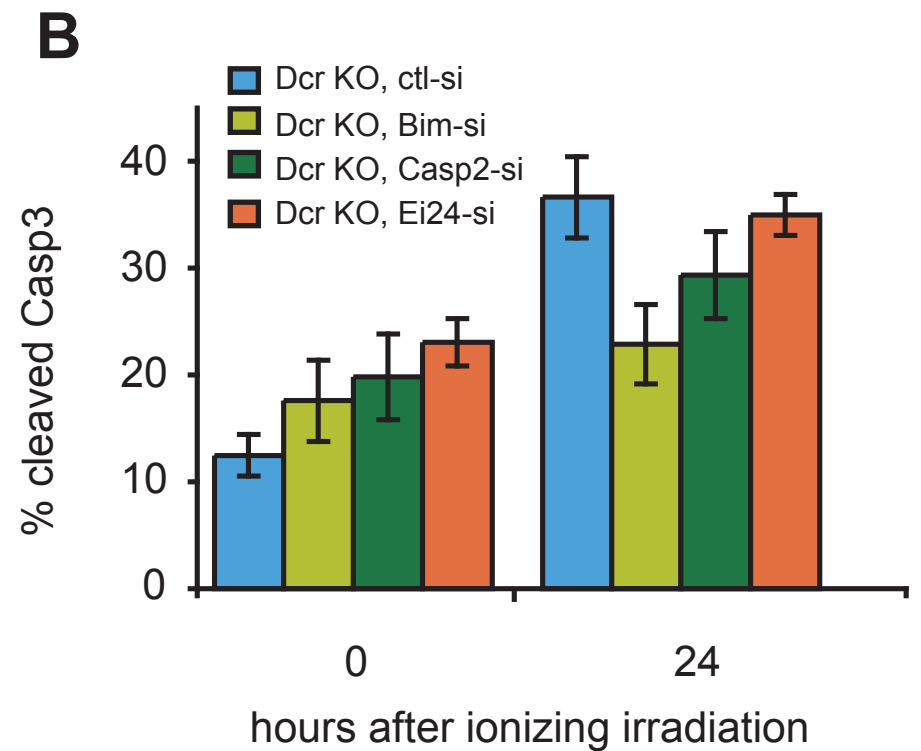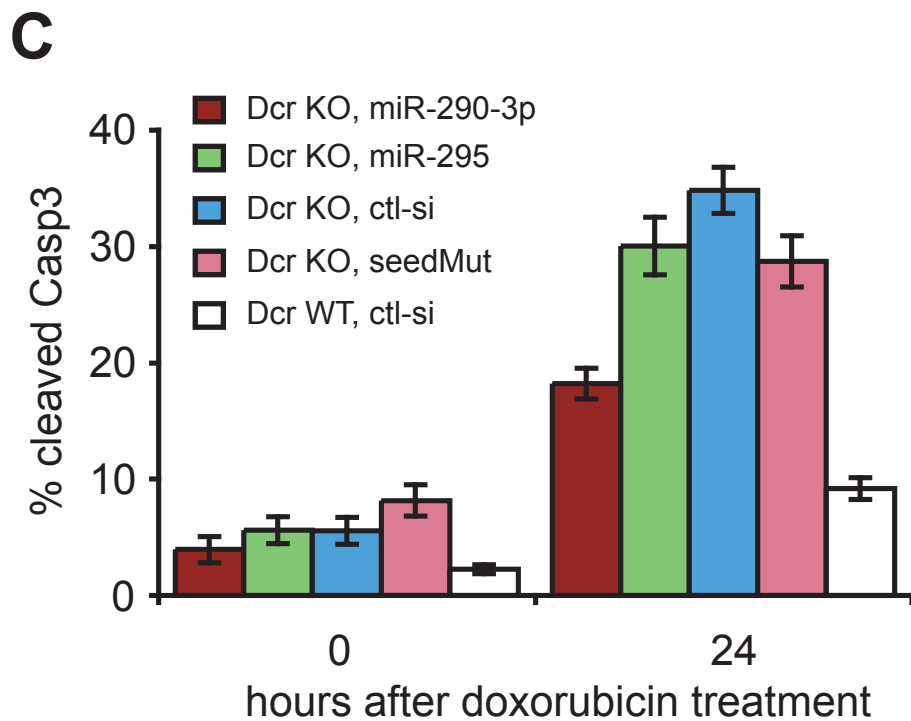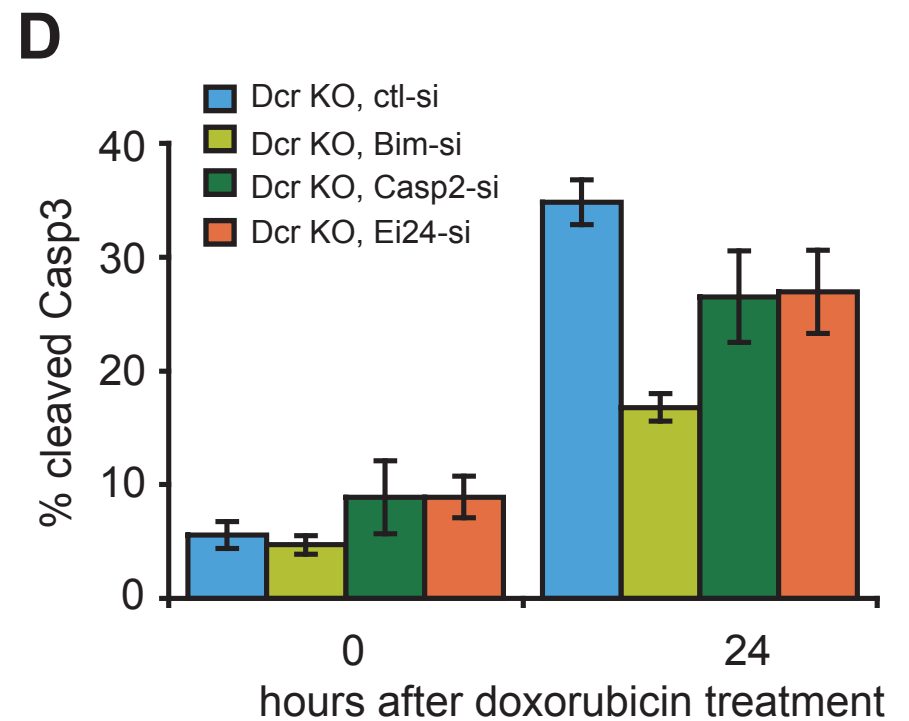

Supplement: Figure S6 — Raw data of Dcr KO and WT cells’ apoptosis response after treatment of 5-Gy radiation or 100 nM doxorubicin. A. Dcr KO cells were treated with 5-Gy radiation 24 hours after transfection of 50 nM miR-295 or miR-290-3p. Caspase 3 activity was assayed 0 and 24 h after the treatment. Transfection of seed mutants and control siRNAs (50 nM) into Dcr KO cells, and overexpression of control siRNAs (50 nM) into WT cells served as controls. B. Dcr KO cells were treated with 5-Gy radiation 24 hours after transfection of 50 nM siRNAs against Bim, Casp2, and Ei24. Caspase-3 activity was assayed 0 and 24 h after the treatment. C,D. A similar series of experiments was performed in Dcr WT and Dcr KO cells using 100 nM doxorubicin. n ≥ 3 for all experiments. Results are shown as mean ± S.E.M. (standard error of the mean). (PDF) [file pgen.1002054.s006.pdf]

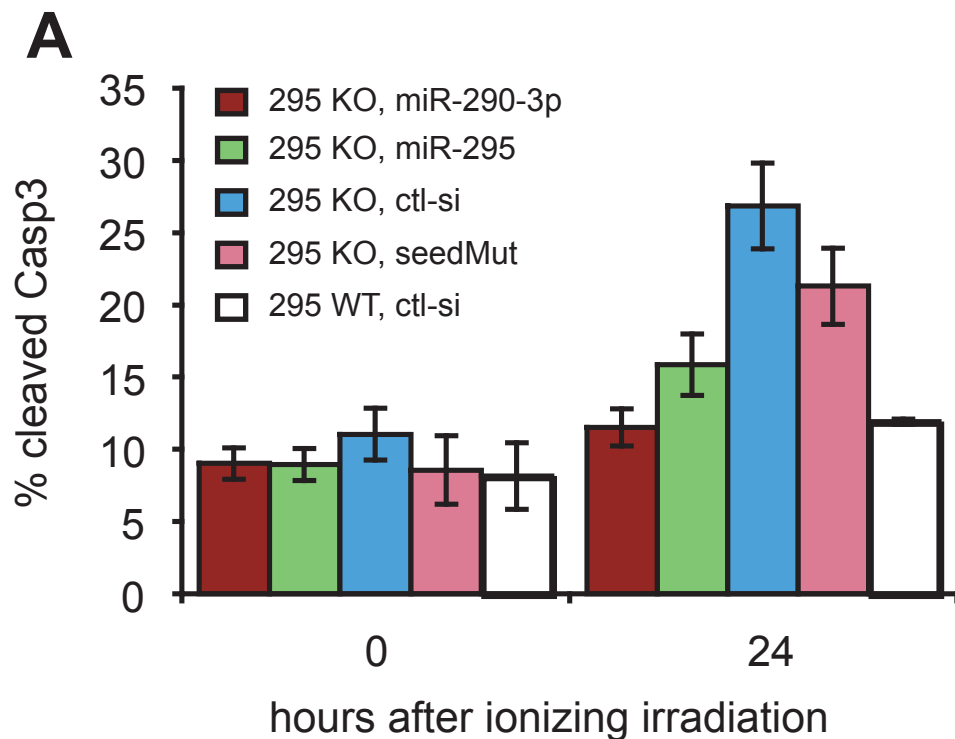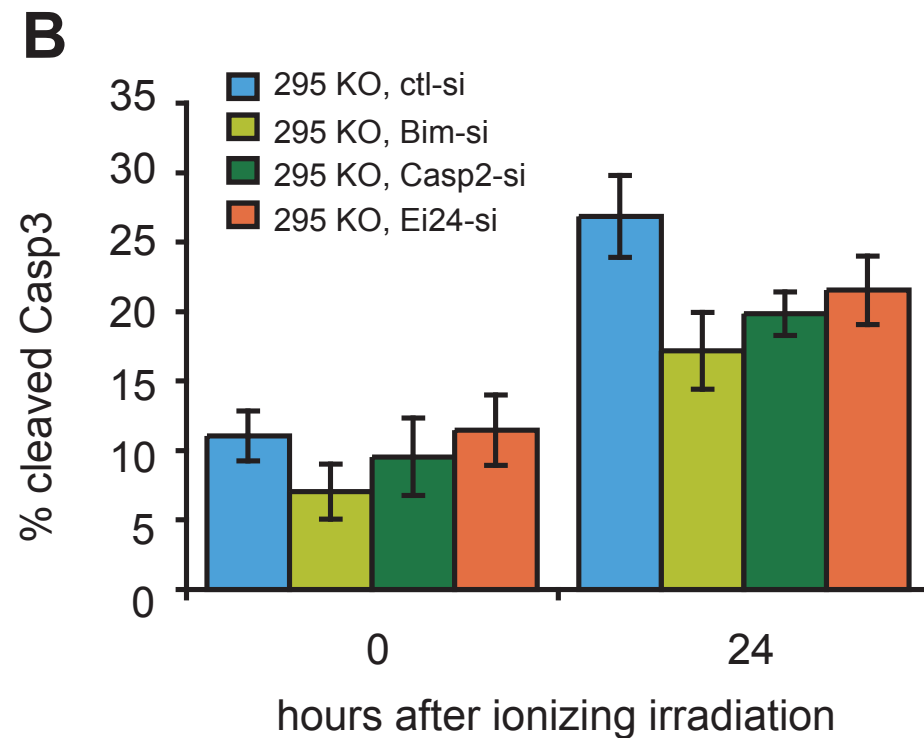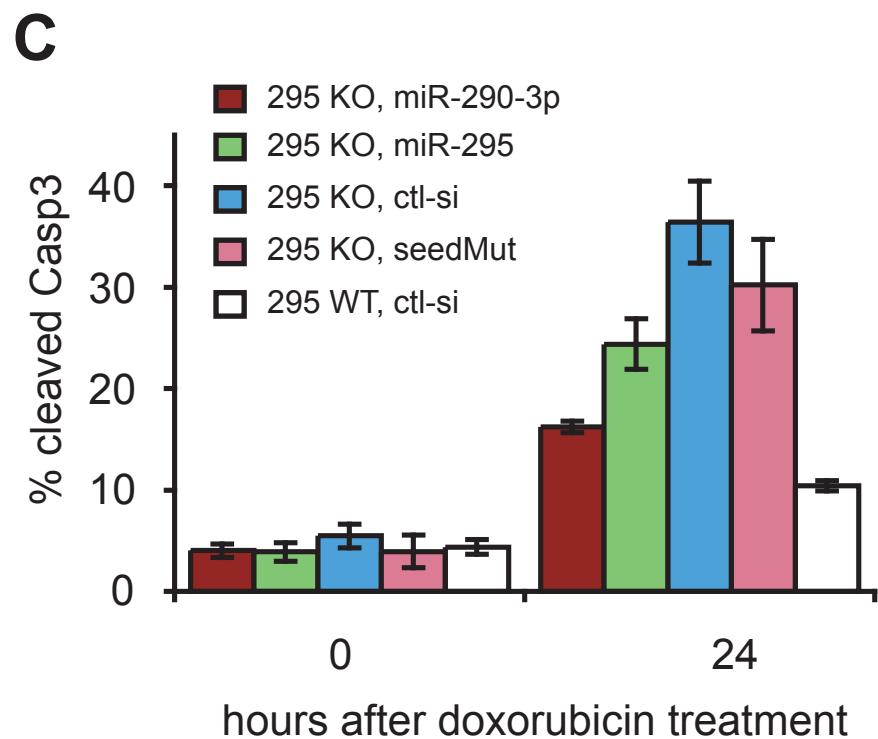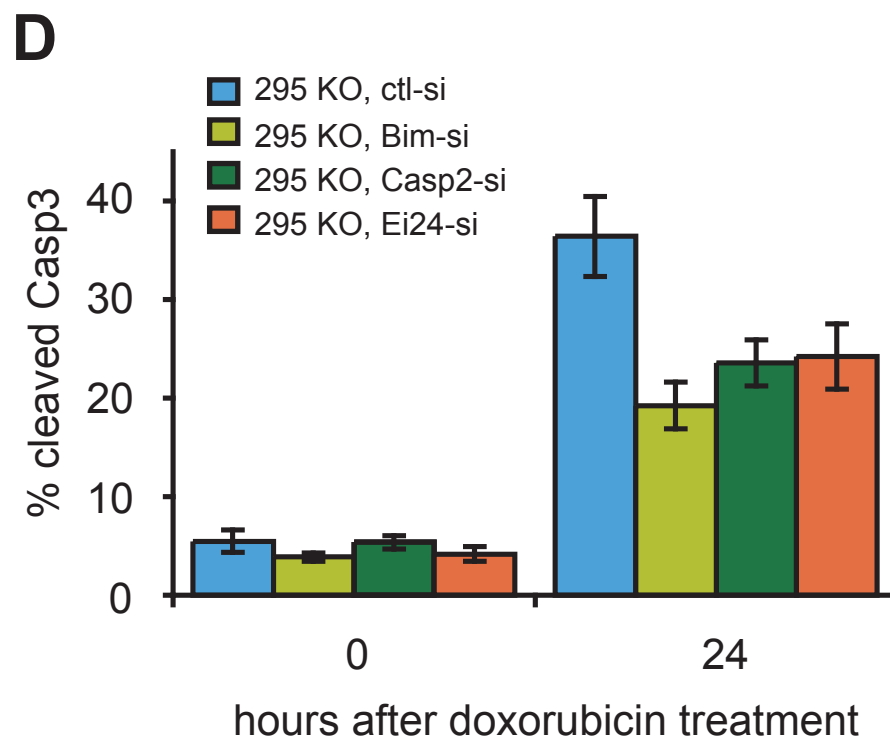

Supplement: Figure S7 — Raw data of 295 KO and 295 WT cells’ apoptosis response after treatment of 5-Gy radiation or 100 nM doxorubicin. A. 295 KO cells were treated with 5-Gy radiation 24 hours after transfection of 50 nM of miR-295 or miR-290-3p. Caspase 3 activity was assayed 0 and 24 h after the treatment. Transfection of seed mutants and control siRNAs (50 nM) into 295 KO cells, and overexpression of control siRNAs (50 nM) into 295 WT cells served as controls. B. 295 KO cells were treated with 5-Gy radiation 24 hours after transfection of 50 nM siRNAs against Bim, Casp2, and Ei24. Caspase-3 activity was assayed 0 and 24 h after the treatment. C,D. A similar series of experiments was performed in 295 WT and 295 KO cells using 100 nM doxorubicin. n ≥3 for all experiments. Results are shown as mean ± S.E.M. (standard error of the mean). (PDF) [file pgen.1002054.s007.pdf]
