# Supplementary material for: A Latent Pro-Survival Function for the Mir-290-295 Cluster in Mouse Embryonic Stem Cells
Source: PLoS Genet. 2011 May 5;7(5):e1002054. doi: 10.1371/journal.pgen.1002054 (PMC3088722; doi:10.1371/journal.pgen.1002054)
Supplement: Table S1 — Sequences and expression level of the mir-295 cluster in ES cells. The 6-mer seed is highlighted in bold. The cloning statistics were taken from previously published studies [7], [8], [9]. (PDF) [file pgen.1002054.s008.pdf]

Cluster	Chr	miRNA	Sequence	% cloned in different ES cell lines		
				Leung et. al. (a)	Babiarz et. al. (b)	Ciaudo et. al. (c)
mir-290-295 cluster	chr7	miR-290-3p	A**AAGUGC**CGCCUAGUUUUAAGCC	0.01	6.31	0.03
		miR-291a-3p	A**AAGUGC**UUCCACUUUGUGUG	1.28	8.36	16.71
		miR-291b-3p	A**AAGUGC**AUCCAUUUUGUUUG	0.03	0.88	0.00
		miR-292-3p	A**AAGUGC**CGCCAGGUUUUGAGUG	2.66	12.47	10.10
		miR-294	A**AAGUGC**UUCCCUUUUGUGUG	13.56	10.82	12.72
		miR-295	A**AAGUGC**UACUACUUUUGAGUC	25.01	13.70	8.89
**Percentage of total miRNA reads**				**42.55**	**52.53**	**48.44**

(a) A.K. Leung, A.G. Young, A Bhutkar, G.X. Zheng, A.D. Bosson, C.B. Nielsen, and P.A. Sharp. Nat Struct Mol Biol. 2011. 18 (2).
(b) J.E. Barbiarz, J.G. Ruby, Y. Wang, D.P. Bartel, and R. Blelloch. Genes Dev 2008. 22 (20).
(c) C. Ciaudo et. al., PLoS Genet. 2009. 5 (8).
