# Supplementary material for: A Latent Pro-Survival Function for the Mir-290-295 Cluster in Mouse Embryonic Stem Cells
Source: PLoS Genet. 2011 May 5;7(5):e1002054. doi: 10.1371/journal.pgen.1002054 (PMC3088722; doi:10.1371/journal.pgen.1002054)
Supplement: Table S2 — Sequences and expression level of the mir-302, mir-467, and mir-17-92 clusters in ES cells. The 6-mer seed is highlighted in bold. The cloning statistics were taken from previously published studies [7], [8], [9]. (PDF) [file pgen.1002054.s009.pdf]

Cluster	Chr	miRNA	Sequence	% cloned in different ES cell lines		
				Leung et. al. (a)	Babiarz et. al. (b)	Ciaudo et. al. (c)
mir-302 cluster	chr3	miR-302a	U**AAGUGC**UUCCAUGUUUUGGUG	1.55	0.14	0.04
		miR-302b	U**AAGUGC**UUCCAUGUUUUAGUA	1.27	0.16	0.00
		miR-302d	U**AAGUGC**UUCCAUGUUUGAGUG	1.63	0.32	0.02
mir-467 cluster	chr2	miR-467a	U**AAGUGC**CUGCAUGUAUAUGC	0.00	0.08	0.33
		miR-467c	U**AAGUGC**GUGCAUGUAUAUGU	0.68	0.00	0.03
		miR-467d	U**AAGUGC**GCGCAUGUAUAUGC	0.21	0.00	0.36
			**Percentage of total miRNA reads**	**5.34**	**0.69**	**0.78**
mir-17 cluster	chr14	miR-17	CA**AAGUGC**UUACAGUGCAGGUA	0.72	1.60	0.45
		miR-20a	UA**AAGUGC**UUAUAGUGCAGGUA	6.44	1.92	0.10
	chrX	miR-20b	CA**AAGUGC**UCAUAGUGCAGGUA	2.04	1.17	0.11
		miR-106a	CA**AAGUGC**UAACAGUGCAGGUA	1.19	0.82	0.12
	chr5	miR-106b	UA**AAGUGC**UGACAGUGCAGA	0.93	0.76	0.17
		miR-93	CA**AAGUGC**UGUUCGUGCAGGUA	0.70	0.83	0.74
			**Percentage of total miRNA reads**	**12.02**	**7.09**	**1.69**

(a) A.K. Leung, A.G. Young, A Bhutkar, G.X. Zheng, A.D. Bosson, C.B. Nielsen, and P.A. Sharp. Nat Struct Mol Biol. 2011.18 (2).
(b) J.E. Barbiarz, J.G. Ruby, Y. Wang, D.P. Bartel, and R. Blelloch. Genes Dev, 2008. 22 (20).
(c) C. Ciaudo et. al., PLoS Genet. 2009. 5 (8).
