# Supplementary material for: A Latent Pro-Survival Function for the Mir-290-295 Cluster in Mouse Embryonic Stem Cells
Source: PLoS Genet. 2011 May 5;7(5):e1002054. doi: 10.1371/journal.pgen.1002054 (PMC3088722; doi:10.1371/journal.pgen.1002054)
Supplement: Table S3 — Predicted targets of the mir-295 cluster. (PDF) [file pgen.1002054.s010.pdf]

Gene symbols of predicted targets
1110018G07Rik
1110021L09Rik
1110037F02Rik
1110067D22Rik
1190002N15Rik
1200009F10Rik
1200015N20Rik
1300001I01Rik
1300014I06Rik
1600029D21Rik
1700021K19Rik
1700061G19Rik
1810013L24Rik
1810026J23Rik
2010301N04Rik
2010305A19Rik
2210010L05Rik
2310008H09Rik
2310035C23Rik
2310044G17Rik
2410014A08Rik
2610020H08Rik
2700078E11Rik
2700081O15Rik
4631416L12Rik
4632434I11Rik
4732496O08Rik
4921505C17Rik
4930453N24Rik
4931414P19Rik
4933434E20Rik
6030458C11Rik
6330409N04Rik
A430093A21Rik
A830007P12Rik
AA881470
Abca1
Abhd12
Abi1
Abl2
Acaa1a
Acbd5

Acox1
Acpp
Adam17
Adam23
Adam9
Adamts5
Aebp2
Agk
AI450540
AI747699
Aifm1
Akt1
Alcam
Aldh1b1
Alg2
Alpk3
Als2cr13
Amotl1
Ampd3
Ank2
Ankfy1
Ankib1
Ankmy2
Ankrd17
Ankrd32
Ankrd44
Ankrd57
Aof1
Ap1g1
Ap1m1
Ap3m1
Apbb2
Apc
Apcdd1
App
Arfgap3
Arhgap1
Arhgap28
Arhgef12
Arhgef17
Arhgef3
Arhgef5
Arhgef7

Arid4a
Arl4a
Arl6ip5
Arpp19
Arsb
Asb8
Ash1l
Asxl2
Atad2
Ate1
Atg16l1
Atg2a
Atp11a
Atp2a2
Atp6ap2
Atp7a
Atrn
AU040829
B230219D22Rik
Bahd1
Bat5
Bbx
BC010304
BC022623
BC038286
BC043118
BC062127
Bcor
Bdh1
Bicd2
Blcap
Blzf1
Bmi1
Bmp2k
Brms1l
Btaf1
Btbd10
Btbd14a
Btbd7
Btbd9
Btg1
Bub3
C330002I19Rik

C330007P06Rik
C77370
C87436
Cadm1
Calm1
Camk1d
Camk2n1
Camsap1
Camsap1l1
Cand1
Casc4
Casd1
Cask
Casp2
Cast
Cav1
Cbara1
Cbx4
Cc2d1a
Ccdc100
Ccdc102a
Ccdc115
Ccdc128
Ccdc93
Ccnd1
Ccnd2
Ccng1
Ccnj
Cd109
Cd151
Cd24a
Cd2ap
Cd55
Cdc23
Cdc2l6
Cdc42se1
Cdh2
Cdk6
Cdkn1a
Cdo1
Cds1
Cds2
Ceacam1

Cenpq
Cep170
Cep55
Cgnl1
Chd3
Chfr
Chic1
Chmp4c
Chn2
Cic
Cirbp
Clcn4-2
Clic1
Clock
Clpb
Clptm1l
Cnot6l
Cobl
Col4a1
Col4a2
Coq10b
Cpd
Cpe
Cpeb1
Cpeb3
Cpne5
Cpt1a
Crebl1
Crebl2
Crem
Crot
Crtc2
Cs
Csf1
Csnk1g1
Cstf2t
Ctgf
Ctsb
Ctxn1
Cxadr
Cyld
Cyp1b1
D030011O10Rik

D030056L22Rik
D10Ertd641e
D12Ertd553e
D16Ertd472e
D2Bwg1335e
D4Wsu53e
D6Wsu163e
D6Wsu176e
D730040F13Rik
D930001I22Rik
Daam1
Dab2
Daglb
Dazap2
Dck
Dcun1d3
Dcun1d4
Ddef1
Ddef2
Ddhd1
Ddx19a
Ddx3x
Ddx46
Ddx5
Dedd
Dennd2c
Derl2
Dgke
Dhdh
Dhx40
Dixdc1
Dnajb5
Dnajb6
Dnajb9
Dnase2a
Dnm1l
Dock4
Dock5
Dock9
Dsg2
Dsn1
Dync1li2
E030018N11Rik

E330016A19Rik
E430028B21Rik
Ect2
Edem1
Edn1
Eea1
EG435970
Egfr
Ehd1
Ei24
Eif2ak2
Eif5b
Elavl2
Elf1
Elk3
Elk4
Enpp4
Entpd4
Entpd7
Epas1
Epc2
Epha2
Epha4
Erbb2ip
Erlin1
Erlin2
Ets1
Etv1
Exo1
Exoc1
Exoc3
Exoc5
Exosc4
F3
Fadd
Fars2
Fbxl3
Fbxo11
Fbxo25
Fbxo39
Fgd4
Fgf4
Fkbp10

Flnc
Fmnl3
Fndc3b
Fosl2
Foxn2
Foxp4
Frmd4a
Fryl
Fusip1
Fut11
Fyco1
Fyn
Fzd6
G3bp2
Gab1
Galnt7
Gcc2
Gfpt2
Ggta1
Gk5
Gnb1l
Gnb5
Gnpnat1
Golga1
Golga2
Gpa33
Gpc6
Gpkow
Gpr107
Gpr177
Gpsm1
Grsf1
Gtf3c2
H13
Hcfc1
Heatr6
Hexim1
Hif1a
Hif1an
Hisppd1
Hmbox1
Hook3
Hoxa1

Hoxd8
Hp1bp3
Hps5
Hrbl
Hs2st1
Hsd17b11
Hspa1b
Ifitm3
Ift80
Igf2bp3
Ikbkb
Il17rd
Il6st
Inhbb
Inoc1
Ints3
Ints6
Irak2
Irak3
Isgf3g
Itch
Itfg1
Itgav
Jmjd3
Jmy
Kbtbd8
Kctd1
Kctd10
Kctd9
Kif16b
Kif1b
Kif3a
Kif5c
Kit
Klhdc5
Klhl20
Klhl28
Klhl7
Ktelc1
Lace1
Lactb2
Lamc1
Lamp2

Laptm4a
Larp4
Lass6
Lats1
Lats2
Lcorl
Leprotl1
Lgals8
Lifr
Lima1
Lmbr1
Lnx2
Lox
Lrig1
Lrrc15
Lrrc58
Lrrc8a
Lycat
Lztfl1
Lztr1
Magi1
Magi3
Man1a
Maoa
Mapt
Marcks
Mastl
Mat2b
Mbd2
Mbnl1
Mbnl2
Mcc
Mdfic
Mdh2
Mecp2
Med28
Med6
Mef2a
Met
Mex3c
Mfap3l
Mfn2
Midn

Mier3
Mink1
Mis12
Mitf
Mkrn3
Mll1
Mllt11
Mllt6
Mmaa
Mmp11
Mmp14
Mmp23
Mn1
Mobkl1a
Mobkl3
Mreg
Mrpl51
Msrb3
Mtbp
Mtm1
Mtmr3
Mtmr4
Mtus1
Mxd1
Myb
Mycn
Mynn
Myo1c
Nagk
Nagpa
Nanos1
Napb
Nbea
Ncam1
Ncapg2
Ncoa3
Ncoa7
Ndn
Ndst1
Necap1
Neo1
Neurod1
Nfatc2ip

Nfib
Nfya
Nhsl1
Niban
Nid1
Nkiras1
Nnat
Nope
Nr2c1
Nr3c1
Nrbp1
Nrp2
Ntn1
Nup35
Oas1g
Obfc2a
Orc2l
Os9
Ostm1
Otub1
Otud4
Otud6b
P2rx4
Pafah1b2
Pank3
Papola
Pbxip1
Pcsk7
Pdgfc
Pdgfd
Pdik1l
Pdpk1
Pdzd2
Pecam1
Pex19
Pfkfb3
Pfn2
Phtf2
Phyhipl
Pigk
Pigm
Pign
Pik3c2a

Pik3cb
Pik3r3
Pja2
Pkd2
Pknox1
Pldn
Plekha3
Plekhm1
Plk4
Plscr3
Pmp22
Polr3f
Ppap2b
Ppp6c
Pqlc1
Prdm4
Prdx3
Prkaa1
Pros1
Ptpn9
Ptprg
Ptprj
Ptprk
Pvr
Qser1
Rab11fip1
Rab21
Rab22a
Rab33b
Rab5c
Rab7l1
Rabgap1
Rad21
Rad23b
Rasa1
Rb1cc1
Rcan1
Reck
Reep3
Rexo2
Rfxdc2
Rgnef
Rhoc

Ripk1
Rnf128
Rnf138
Rnf14
Rnf146
Rnf20
Rock2
Rpia
Rragd
Rreb1
Rrm2
Rrm2b
Rtn1
Rtn4
Rtn4rl1
Sall1
Sart3
Sass6
Sbf1
Scoc
Scp2
Scpep1
Sdc1
Sdc2
Sema3c
Sema4b
Senp7
Sephs1
Serinc1
Serinc3
Serpinb9b
Serpine1
Serpini1
Sertad2
Setbp1
Sgms1
Sh3gl2
Shoc2
Sipa1l3
Sirt7
Skp2
Slain2
Slc10a7

Slc12a2
Slc12a7
Slc16a6
Slc16a9
Slc19a2
Slc25a16
Slc25a40
Slc30a1
Slc35a4
Slc35a5
Slc35b4
Slc39a6
Slc4a4
Slc6a15
Slc6a6
Slco5a1
Slu7
Smad2
Smad6
Smarcc2
Smg7
Smndc1
Snap29
Sntb2
Snx12
Snx16
Snx30
Socs5
Sod2
Sos1
Sox11
Sox21
Sparc
Spin4
Spnb2
Spp1
Spry4
Sptlc1
Spty2d1
Ssbp2
Ssfa2
Ssr1
Ssx2ip

St3gal5
St5
Stambp
Stard13
Stat1
Stk11ip
Stk3
Stk38
Stk38l
Stk4
Strn
Styx
Sumf1
Surf4
Susd2
Suv39h1
Suv420h1
Syap1
Syncrip
Synj2
Taf7
Taf9b
Tanc1
Taok1
Tapt1
Tbc1d12
Tbc1d15
Tbc1d23
Tbc1d8b
Tbc1d9
Tbcel
Tbl1xr1
Tbx3
Tceb3
Tcfap4
Tcfcp2l1
Tfb2m
Tfrc
Tgfbr1
Tgfbr2
Tgoln1
Thbs1
Thtpa

Tiam1
Tinagl
Tiparp
Tle4
Tm9sf3
Tmco1
Tmed2
Tmed4
Tmem111
Tmem123
Tmem127
Tmem16f
Tmem184b
Tmem188
Tmem33
Tmem41b
Tmem43
Tmem64
Tmem79
Tmem87b
Tmtc3
Tmub2
Tnfaip1
Tnfrsf21
Tnrc18
Tnrc6b
Tnrc6c
Tob2
Tom1l2
Topors
Tor1b
Tpk1
Tpm2
Tpp1
Trak1
Trib1
Trim25
Trim35
Trim36
Trps1
Tsga14
Tspyl2
Tsr2

Ttc3
Ttc30b
Ttc8
Tubb2b
Tulp4
Twf1
Txnrd3
Tyw3
Ube2g1
Ube2q2
Ube2w
Ube2z
Ube4a
Ubfd1
Ubtf
Ubxd4
Ubxd6
Uchl1
Unkl
Usp25
Usp33
Usp42
Usp46
Usp9x
Vcl
Vim
Vps26a
Vps4b
Wars2
Wdr1
Wdr40b
Wdr42a
Wdr48
Wee1
Wisp1
Yipf6
Ythdf3
Ywhaz
Zbtb4
Zbtb41
Zcchc2
Zdhhc20
Zdhhc9

Zfand2a
Zfhx3
Zfp11
Zfp113
Zfp148
Zfp180
Zfp191
Zfp213
Zfp238
Zfp296
Zfp319
Zfp326
Zfp408
Zfp422
Zfp46
Zfp53
Zfp553
Zfp568
Zfp597
Zfp650
Zfp668
Zfp697
Zfp704
Zfp74
Zfp790
Zfp800
Zfp84
Zfp91
Zfx
Zfyve26
Zmpste24
Zmym3
Znfx1
Zwint
