# Supplementary material for: A Latent Pro-Survival Function for the Mir-290-295 Cluster in Mouse Embryonic Stem Cells
Source: PLoS Genet. 2011 May 5;7(5):e1002054. doi: 10.1371/journal.pgen.1002054 (PMC3088722; doi:10.1371/journal.pgen.1002054)
Supplement: Table S4 — Oligos and siRNAs used in all experiments. (PDF) [file pgen.1002054.s011.pdf]

**siRNA**
miR-295
miR-295<br>seed mutant
miR-290-3p
Control siRNA
si-bim
si-casp2
si-ei24
**RT-PCR Primers**
Casp2 Forward
Casp2 Reverse
Ei24 Forward
Ei24 Reverse
β-actin Forward
β-actin Reverse
**3′UTR Primers**
Irf9 Forward
Irf9 Reverse
Casp2 Forward
Casp2 Reverse
Pax6 Forward
Pax6 Reverse
p21 Forward
p21 Reverse
Thbs1 Forward
Thbs1 Reverse
Itgav Forward
Itgav Reverse
Irak3 Forward
Irak3 Reverse
Lats2 Forward
Lats2 Reverse
**Mutagenic Primers**
Ei24 Site 1
Casp2 Site 1
Casp2 Site 2
Casp2 Site 3
Casp2 Site 4
**Northern probes**
miR-292 probe
miR-295 LNA

Sequence (5′ - 3′ unless otherwise noted)
5′- AAAGUGCUACUACUUUUGAGUCU -3′<br>3′- UCUUUCACGAUGAUGAAAACUCA -3′
5′- AAAGACGUACUACUUUUGAGUCU -3′<br>3′- UCUUUCUGCAUGAUGAAAACUCA -3′
5′- AAAGUGCCGCCUAGUUUUAAGCCC -3′<br>3′- CCUUUCACGGCGGAUCAAAAUUCG -5′
(from Dharmacon, Accell Non-targeting pool)
(from Dharmacon, Smartpool)
(from Dharmacon, Smartpool)
(from Dharmacon, Smartpool)
GCAGGGTCACTTGGAAGACT
GAAGACAGGGAGGACCATCA
TCTCTTCCCCATCCATCTT
TAACGTAACGACACTCCTTTC
GACGAGGCCCAGAGCAAGAGAGG
GGTGTTGAAGGTCTCAAACATG
AATAACTCGAGCGCGTCTCCATGGAAATAGA
AATAAGGGCCCTTTAATTTGGAGCTCACATTTCT
AATAACTCGAGCCGCCTGCTATTCCTGCT
AATAAGGGCCCTCAACATTTATTTGGCACCTG
AATAACTCGAGAGAGAGAAGGAGAGAGCATGTG
AATAAGGGCCCAAATCATTCTGAGGATTTCTAGGG
AATAACTCGAGCCTCTTCTGCTGTGGGTCA
AATAAGCGGCCGCAATCATCGAGAAGTATTTATTGAGC
AATAACTCGAGTCATCAGCTGCCAATCATAA
AATAAGGGCCCTTCCATATGATTTATTGTTGTTCCTT
AATAACTCGAGCCACTTCTGTCCGCTCCA
AATAAGGGCCCGAAGTCAACTGTAGTGTAATGTGTACC
AATAACTCGAGATCCACCAGAAGATCAAGCAA
AATAAGCGGCCGCTTTTATATAACAATTGGAATGCCACAG
AAACTCGAGCGAGGAAACCCAAAATGAGA
AAAGGGCCCTCCAACAAAACACCACAAATG
GACCAGAGTTTTCCAGCTGTTTTTTACGTCTTGCCAGCTCCTGT
CCTTACTGTGGCTTCTGCATCGTCTTACACTGTACTTGACGGC
GTACCATATGTGATATAACCTAGAACGTCTTGTCTCTGCTCTTATGAAACTTG
GTGCTTACTGCAGGCTGTAATGCGTCTTTTGCTTGTTTCACTTGTTC
CTTACTTACTGATATCCAGTAACTGCGTCTTACTAGGTCTTCATGAATGTTTC
CAAAAGAGCCCCCAGTTTGAGT
AGACTCAAAAGTAGTAGCACTTT
